# The association between obesity related adipokines and risk of breast cancer: a meta-analysis

**DOI:** 10.18632/oncotarget.17853

**Published:** 2017-05-13

**Authors:** Yu Gui, Qinwen Pan, Xianchun Chen, Shuman Xu, Xiangdong Luo, Li Chen

**Affiliations:** ^1^ Breast Disease Center, Southwest Hospital, Third Military Medical University, Chongqing, China; ^2^ Burn Research Institute, Southwest Hospital, Third Military Medical University, Chongqing, China; ^3^ National Key Laboratory of Trauma and Burns, Chongqing Key Laboratory of Disease Proteomics, Chongqing, China

**Keywords:** obesity, adipokine, breast cancer

## Abstract

The risk of breast cancer is significantly increased among obese women as the deleterious adipokines can be over secreted and beneficial adipokines can be hyposecreted. We aim to evaluate the association between obesity-associated adipokines and breast cancer. We searched PubMed, EMBASE, Web of Science, and Chinese Biomedical Literature (CBM) databases for studies reporting association of obesity related adipokines with breast cancer published before Sept. 15, 2015. Initially, 26783 publications were identified, and later, 119 articles were selected for further meta-analysis. Out of these 119 studies, twenty-six studies had reported adipokine levels among obese and non-obese healthy subjects and ninety-three studies had reported adipokine levels among patients with breast cancer. The subjects with BMI >25 kg/m2 had significantly lower adiponectin levels and higher leptin and tumor necrosis factor-α (TNF-α) levels than those with BMI <25 kg/m2. Decreased concentrations of adiponectin, and increased concentrations of leptin, IL-6, IL-8, TNF-α, resistin and visfatin were significantly associated with risk of breast cancer. Adipokine levels were strongly associated with breast cancer among Asian women as compared to non-Asian women. Our results might explain the relationship of obesity, adipokine levels and risk of breast cancer, especially in Asian women.

## INTRODUCTION

Breast cancer is the most common cancer across the world. Approximately, 508,000 females died due to breast cancer in 2011 world over [1]. Breast cancer is now a disease of both the developed and developing countries. Obesity is an important modifiable risk factor for breast cancer. Current WHO data shows that worldwide obesity has more than doubled since 1980 [2]. About 39% of the global population is overweight or obese, with higher rates seen among women [2]. Obesity has been an increasing public health problem for the past 30 years; and currently almost all nations are affected by this health disorder. Obesity is considered an important risk factor for many serious diseases, including diabetes mellitus, metabolic syndrome, cardiovascular diseases, and certain cancers [3]. During the past decade an explosion of evidence has linked obesity with increases in the incidence of cancer and associated mortality [4]. Overweight/obesity is also an important risk factor for breast cancer among postmenopausal women [5]. Excess body weight significantly increases the risk of postmenopausal breast cancer risk by 30%–50% [6]. Obesity is also associated with increased tumor burden and histo-pathological grade, and a higher incidence of lymph node metastasis among breast cancer patients [4]. The mechanisms by which obesity contributes to breast cancer are complex and have not yet been fully elucidated. Several reports have shown that abnormal levels of estrogen, insulin or adipokines may be responsible for the increased risk of breast cancer among obese women [7, 8].

Adipokines, small peptide hormonal growth factors, are mainly secreted by adipocytes of white adipose tissue. Breast tissue mainly comprises of adipocytes (almost 90%), and epithelial cells (rest 10% of the breast volume) [9]. Over-secretion of deleterious pro-inflammatory adipokines such as leptin, tumor necrosis factor-α (TNF-α), interleukin-6 (IL-6), interleukin-8 (IL-8), plasminogen activator inhibitor-1 (PAI-1), resistin, hepatocyte growth factor (HGF), and hyposecretion of beneficial adipokines like adiponectin and visfatin has been reported among obese persons [10, 11]. The dysfunction of adipokine pathways is considered as an important cause of obesity induced disease [10, 11]. Recently, there has been a considerable interest in the potential role of adipokines in the development of breast cancer [12]. Vona-Davis et al. also demonstrated that adipokines are the major contributing factors for obesity associated breast cancer, and recent meta-analyses have shown that adiponectin levels are lower in breast cancer patients [13, 14].

Although over twenty adipokines are known; only twelve (adiponectin, leptin, IL-6, TNF-α, HGF, PAI–1, resistin, secreted frizzled-related protein 5 (SFRP-5), lipocalin 2, IL-8, apelin and visfatin) have been implicated in breast cancer [15]. With the aim to gain a better insight into the relationship between obesity and breast cancer risk, we sought to clarify the contribution of individual adipokines (adiponectin, leptin, and IL-6 *etc.*) to obesity, and to provide a reference for breast cancer biomarkers. We conducted a meta-analysis to investigate the association between adipokines, obesity and risk of breast cancer.

## RESULTS

### Characteristics of identified studies

Initially, the database search identified a total of 26783 studies. After evaluating the titles and abstracts, 26509 studies were excluded. Subsequently, 274 potentially relevant full text articles were reviewed further. Among these, 155 were excluded for the following reasons: seventy-nine studies had no or insufficient data, nine had same or overlapping data, thirteen studies had detected adipokine levels in breast tissue, thirty-eight articles were reviews, commentaries or letters, five studies included male participants, three articles had conducted *in-vitro* estimation, and additional eight studies were excluded because of unavailability of full text. Finally, 119 were selected for meta-analysis, twenty-six studies had compared levels of adipokines among obese and non-obese healthy subjects and ninety-three studies had evaluated among patients with breast cancer. Supplementary Figure 1 shows the flow of the literature search and selection criteria.

The general characteristics of participants from all the included studies are presented in Supplementary Table 1 and Supplementary Table 2. A total of 3787 cases and 5231 controls had been included in studies investigating the association of adipokines and obesity among obese and non-obese subjects; 12,301 cases and 12,805 controls had investigated the adipokines levels and breast cancer risk. Twenty-six studies (including 23 case-control studies and 3 cross-sectional studies) had reported adipokine levels among obese and non-obese healthy subjects; 11, 16, 7, 9, 4, 2 and 1 studies had analyzed the levels of adiponectin, leptin, IL-6, TNF-α, resistin, visfatin and PAI-1, respectively. Of the 93 studies (including 85 case-control studies, 2 cross-sectional studies and 6 cohort studies) reporting levels of adipokines levels among patients with breast cancer, 46 had evaluated leptin, 29 had evaluated adiponectin, 29 had estimated IL-6 levels, 17 had estimated TNF-α, six had evaluated resistin, and lastly, three each had evaluated HGF, PAI-1, and visfatin. The main adjustments in each individual study were BMI, adiponectin, leptin, resisitin and visfatin (Supplementary Table 2). The quality scores of all studies were at least 6 (Supplementary Table 1 and Supplementary Table 2).

### Global analysis of adipokines on obese and non-obese subjects

**Figure 1:**
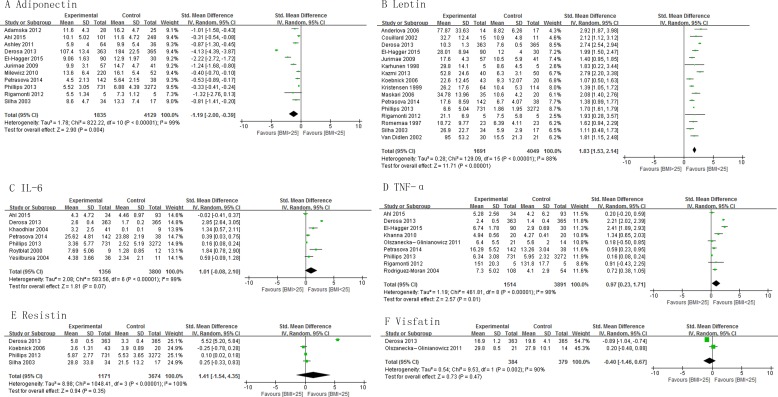
Association of adiponectin, leptin, IL-6, TNF-α, resistin and visfatin with BMI

This meta-analysis demonstrated that the levels of adiponectin were significantly lower in the subjects with BMI >25 kg/m^2^ than those with BMI < 25 kg/m^2^ , pooled SMD -1.19 (95% CI, -2.00, -0.39; *P* = 0.004) (Figure 1A). The subjects with BMI > 25 kg/m^2^ had significantly higher concentrations of leptin and TNF-α than those with BMI < 25 kg/m^2^, pooled SMD of 1.83 (95% CI, 1.53, 2.14; *P* < 0.00001) and 1.97 (95% CI, 0.23, 1.71; *P* = 0.01), respectively (Figure 1B and 1D). There was no significant difference in the levels of IL-6, resistin and visfatin between subjects with BMI > 25 kg/m^2^ and those with BMI < 25 kg/m^2^ (Figure 1C, 1E and 1F). The subjects with BMI > 30 kg/m^2^ had significantly lower concentrations of adiponectin than those with BMI < 30 kg/m^2^ and the levels of leptin and TNF-α were significantly higher in the subjects with BMI >30 kg/m^2^ than those with BMI < 30 kg/m^2^ (Supplementary Figure 2). Subgroup analysis on basis of study design shows similar results among cross-sectional and case-control studies for adiponectin, leptin, IL-6, and TNF-α levels (Supplementary Figure 3).

### Global analysis of adipokines on breast cancer occurrence

**Figure 2:**
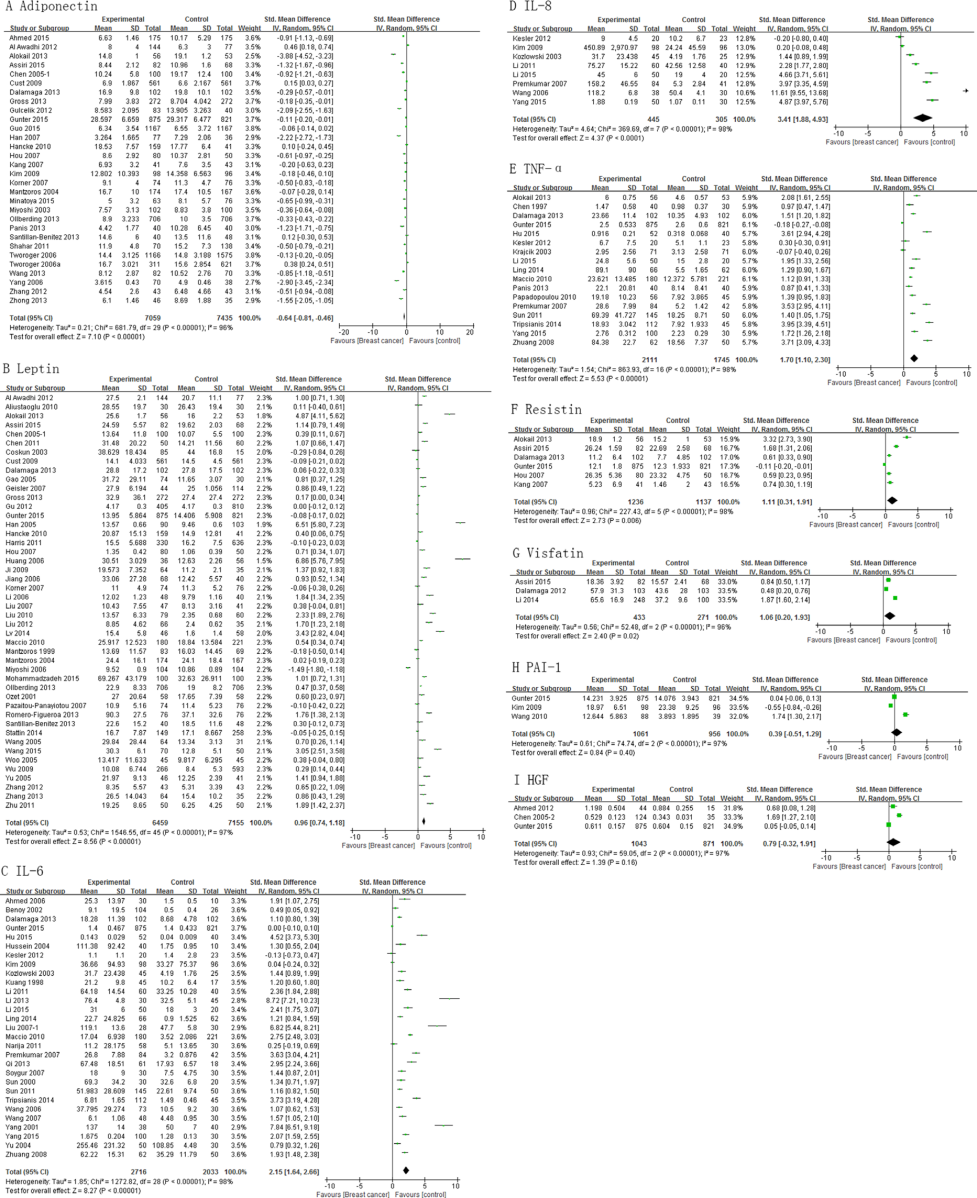
Association of adiponectin, leptin, IL-6, IL-8, TNF-α, resistin, visfatin, PAI-1 and HGF with breast cancer risk

Analysis shows that the cases had significantly lower levels of adiponectin than controls , pooled SMD of -0.64 (95% CI, -0.81, -0.46; *P* < 0.00001) (Figure 2A). Mean concentrations of leptin, IL-6, IL-8, TNF-α, resistin, and visfatin were higher in cases than controls (pooled SMD 0.96 (95% CI, 0.74, 1.18; *P* < 0.00001), 2.15 (95% CI, 1.64, 2.66; *P* < 0.00001), 3.41 (95% CI, 1.88, 4.93; *P* < 0.0001), 1.70 (95% CI, 1.10, 2.30; *P* < 0.00001), 1.11 (95% CI, 0.31, 1.91; *P* = 0.006), and 1.06 (95% CI, 0.20, 1.93; *P* = 0.02), respectively (Figure 2B–2G). However, PAI-1 and HGF levels did not differ significantly between cases and controls (Figure 2H and 2I). Subgroup analysis for adiponectin and leptin on basis of study design showed consistent results among cross-sectional and case-control studies, except cohort studies (Supplementary Figure 4).

### Influence of race on levels of adipokines

**Figure 3:**
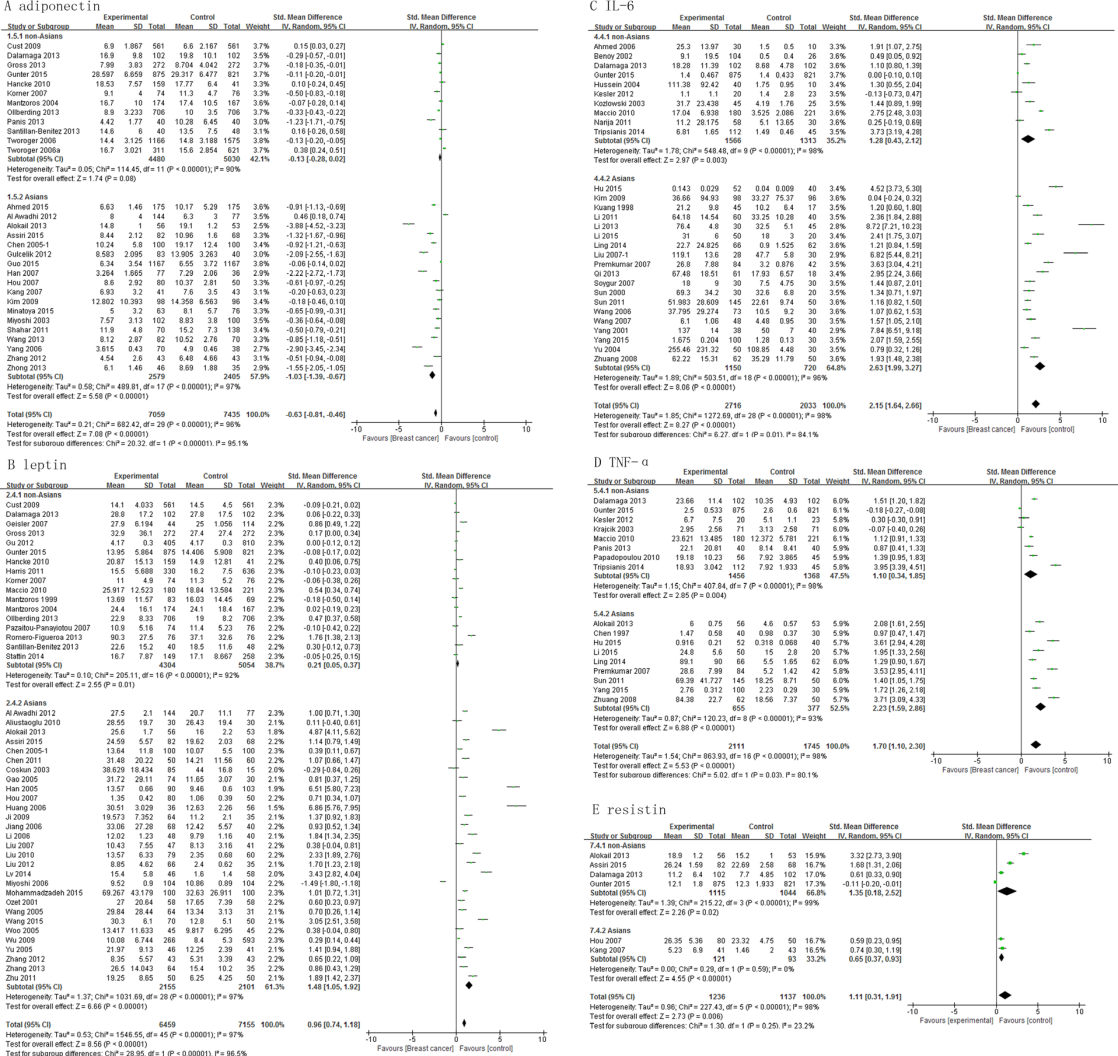
Association of adiponectin, leptin, IL-6, TNF-α and resistin with breast cancer risk by population characteristics subtype

A correlation could be observed between the levels of adipokines and incidence of breast cancer among Asians. The adiponectin levels of Asian cases were lower than the Asian controls, with a pooled SMD -1.03 (95% CI, -1.39, -0.67; *P* < 0.00001), but this association was not significant among non-Asians (SMD, -0.13; 95% CI, -0.28, 0.02; *P* = 0.08) (Figure 3A). Both Asian and non-Asian cases had significantly higher leptin levels than controls, with a pooled SMD of 0.21 (95% CI, 0.05, 0.37; *P* = 0.01) and 1.48 (95% CI, 1.05, 1.92; *P* < 0.00001), respectively (Figure 3B). Similar association was found for IL-6 and TNF-α levels (Figure 3C and 3D). The resistin levels were significantly higher among Asian cases than Asian controls with pooled SMD of 1.57 (95%CI, 0.53 - 2.60; *P* = 0.003), but resistin levels did not differ significantly between non-Asian cases and controls (Figure 3E).

### Influence of sample types and detection method

**Figure 4:**
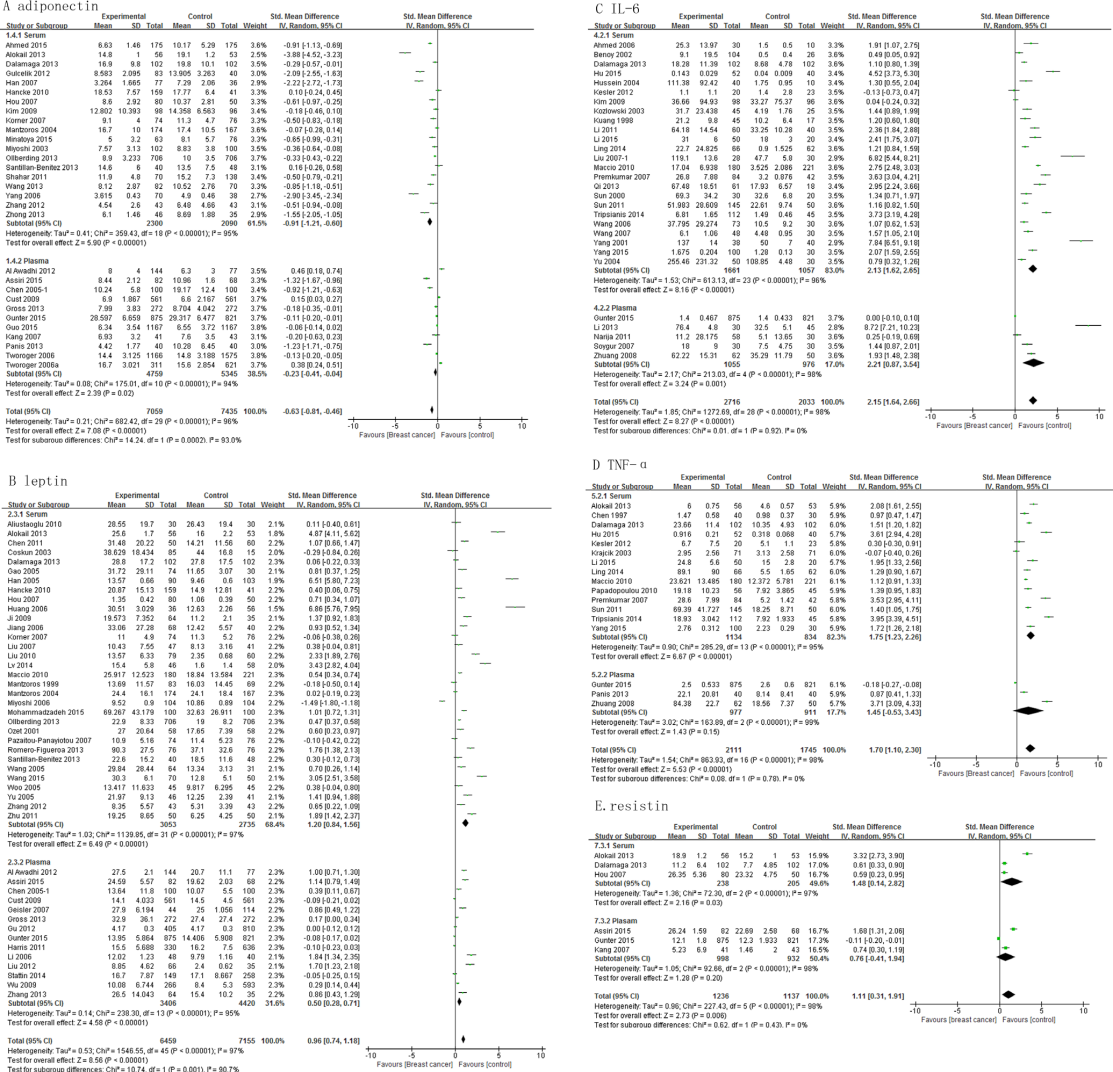
Association of adiponectin, leptin, IL-6, TNF-α and resistin with breast cancer risk by sample types

While evaluating the relevance of sampling, our results show that serum levels of adiponectin, leptin, IL-6, TNF-α and resistin differed significantly between cases and controls, with a pooled SMD of -0.91 (95% CI, -1.21, -0.60; *P* < 0.00001), 1.20 (95% CI, 0.84, 1.56; *P* < 0.00001), 2.13 (95% CI, 1.62, 2.65; *P* < 0.00001), 1.75 (95% CI, 1.23, 2.26; *P* < 0.00001), and 1.48 (95% CI, 0.14, 2.82; *P* = 0.03), respectively (Figure 4). On the other hand, only plasma levels of adiponectin (pooled SMD, -0.23; 95% CI, -0.41, -0.04; *P* = 0.02), leptin (pooled SMD, 0.50; 95% CI, 0.28, 0.71; *P* < 0.00001) and IL-6 (pooled SMD, 2.21; 95% CI, 0.87, 3.54; *P* = 0.001) (Figure 4A–4C) differed significantly. Plasma levels of TNF-α and resistin did not differ significantly between cases and controls (Figure 4D–4E).

Levels of adiponectin, leptin, TNF-α and IL-6 detected by ELISA differed significantly between cases and controls, with a pooled SMD of -0.70 (95% CI, -0.93, -0.47; *P* < 0.00001), 0.56 (95% CI, 0.33, 0.82; *P* < 0.0001), 2.06 (95% CI, 1.56, 2.56; *P* < 0.00001) and 1.95 (95% CI, 1.36, 2.54; *P* < 0.00001), respectively (Supplementary Figure 5A–5D). Detection of mean adipokine levels by Radioimmunoassay (RIA) indicated significant differences between cases and controls, with a pooled SMD of -0.15 (95% CI, -0.42, 0.11; P=0.26), 1.28 (95% CI, 0.87, 1.69; *P* < 0.00001), 4.05 (95% CI, 1.69, 6.41; *P* = 0.0008) and 1.41 (95% CI, 1.13, 1.70; *P* < 0.00001), respectively (Supplementary Figure 5A–5D), but these associations were not significant when Multiplex assay was used.

### Association of adipokines and clinical parameters in breast cancer

ER positive cases had significantly higher leptin levels than ER negative cases (SMD, 0.65; 95% CI, 0.02, 1.28; *P* = 0.04). Adiponectin levels were significantly lower among patients with TNM III and IV stage cancer as compared to patients of TNM I and II stage cancer (pooled SMD 0.67, 95% CI, 0.36, 0.97, *P* < 0.0001). On the other hand, the mean concentration of leptin, IL-6, TNF-α and HGF were significantly higher in TNM III/IV stage patients than those of TNM I/II stage (pooled SMD -1.51, 95% CI, -2.41, -0.60, *P* = 0.001; -1.38, 95% CI, -1.72, -1.04, *P* < 0.00001; -1.38, 95% CI, -2.39, -0.36, *P* = 0.008; and -6.94, 95% CI, -7.89, -5.99, *P* < 0.00001, respectively). Leptin and TNF-α levels were also significantly higher in lymph node metastasis (LNM) positive cases than in LNM negative cases, with a pooled SMD of 0.80 (95% CI, 0.45, 1.14; *P* < 0.00001) and 0.63 (95% CI, 0.30, 0.96; *P* = 0.0002) respectively. Leptin levels were significantly higher among postmenopausal cases than premenopausal cases (Table 1).

**Table 1:** Association of adipokines with breast cancer risk by clinical parameters

Comparison	No. of Studies	SMD	95%CI	*P* value
**ER (+ VS −)**				
Leptin	3	0.65	0.02, 1.28	0.04
**TNM (I+II VS III+IV)**				
Adiponectin	3	0.67	0.36, 0.97	< 0.0001
Leptin	5	−1.51	−2.41, −0.60	0.001
IL-6	9	−1.38	−1.72, −1.04	< 0.00001
TNF-ɑ	4	−1.38	−2.39, −0.36	0.008
**LNM (+ VS −)**				
Adiponectin	3	−0.54	−1.41,0.33	0.23
Leptin	7	0.80	0.45, 1.14	< 0.00001
IL-6	3	−0.43	−1.66, 0.80	0.50
TNF-ɑ	3	0.63	0.30, 0.96	0.0002
**Menopausal status (Pre VS Post)**				
Adiponectin	8	0.14	−0.31, 0.59	0.54
Leptin	10	−0.73	−1.17, −0.29	0.001
Resistin	3	−0.05	−1.17, 1.07	0.93

Heterogeneity tests indicated a significant heterogeneity among studies with *I*^2^ > 70% (Figure 2). Heterogeneity was significantly reduced when studies were grouped by detection method (with *I*^2^ = 0%) (Supplementary Figure 5D). We also observed that heterogeneity was markedly reduced after grouping of studies by menopausal status (*I*^2^ = 0%) (Supplementary Figure 6C), suggesting that the classification of detection method and menopausal status might contribute to heterogeneity. Publication bias was detected by funnel plots (Supplementary Figure 7), suggesting some evidence of publication bias.

## DISCUSSION

This meta-analysis summarized evidence for levels of adipokines among obese and non-obese healthy subjects and the relationship between circulating adipokines and risk of breast cancer. Our findings indicate that obese subjects had lower adiponectin levels and higher leptin and TNF-α levels than non-obese subjects. We also found that decreased circulating adiponectin levels and increased leptin, IL-6, IL-8, TNF-α, resistin and visfatin levels are significantly associated with risk of breast cancer. The levels of adipokines among Asian women were more closely associated with risk of breast cancer than non-Asians. Serum concentrations of adipokines were associated with risk of breast cancer, whereas plasma concentrations of TNF-α and resistin did not differ significantly between cases and controls. Estimation by ELISA showed a significant difference between the levels of adipokines in the cases and controls, however, this difference was non-significant upon detection by Multiplex assay. The levels of adiponectin, leptin, IL-6 and TNF-α were significantly associated with higher TNM classification, and leptin levels were associated with ER and LNM positive stages and TNF-α levels were associated with LNM status.

In addition to the patients with breast cancer, levels of adipokines were also evaluated among obese subjects. In comparison to non-obese subjects, circulating leptin and TNF-α levels were higher, and adiponectin levels were lower among obese persons (Figure 1). This pattern is similar to that observed among breast cancer patients. This indicates that obese women are probably at a higher risk of breast cancer. The underlying pathology might be related to the endocrine and metabolic profile of patients with breast cancer and obese subjects. Accumulating evidence in recent years has demonstrated that the increased production and secretion of a wide range of adipokines from breast adipocytes among obese patients might have a profound effect on tumor progression [16].

In this meta-analysis we found that patients with breast cancer had significantly lower adiponectin levels than controls. It is known that adiponectin not only possesses anti-atherosclerotic, anti-inflammatory, and insulin-sensitizing properties, but it also has protective effect against cancer [17, 18]. Bariatric surgeries among obese women have demonstrated that an average weight loss from 14% to 25% can significantly improve adiponectin levels and reduce the risk of breast cancer [19]. Therefore, high adiponectin levels are significantly associated with risk of breast cancer which is consistent with results of previous meta-analyses [14, 20, 21]. As compared to the previous meta-analyses, our meta-analysis included more studies and directly calculated the summary statistic by use of the mean and SD values of adiponectin. We also found high leptin levels among breast cancer cases. Higher serum levels of leptin have also been reported previously among obese cancer patients as compared to women with normal weight [22]. Previous studies have reported that levels of leptin were not only correlated with tumor size but also with tumor hormonal receptor status (ER and progesterone receptor (PR)) and were significantly higher in breast cancer patients than healthy controls [23, 24]. Leptin promotes angiogenesis and pro-inflammatory responses; and stimulates the proliferation of normal and malignant breast epithelial cells [25, 26]. *In-vitro* studies indicate that leptin influences different intracellular signaling pathways including mitogen-activated protein kinase (MAPK), Janus kinase 2–signal transducer and activator of transcription 3 (JAK2–STAT3) and phosphatidylinositol 3-kinase–protein kinase B (PI3K–AKT) [27]. In this study, we also confirmed that leptin levels were significantly related to the occurrence of breast cancer, which is consistent with previous studies. Similar to the results of earlier analyses [28], our results also show that leptin levels are associated with increased risk of breast cancer. Our meta-analysis included 46 articles for leptin, however, Niu et al. had reported with only 14 articles.

TNF-α was originally identified as a polypeptide cytokine secreted by macrophages that infiltrate the adipose tissue. Plasma TNF-α concentration is positively correlated with BMI [29]. Clinical studies have reported that the increased levels of TNF-α mRNA among overweight/obese subjects are associated with increased risk of breast cancer risk and TNF-α expression in the peripheral blood mononuclear cells (PBMC) of obese subjects decreases after reduction in weight [30]. Previous studies have found increased concentrations of TNF-α in the breast tumor cytosol, and, presence of TNF-α has been strongly correlated with metastatic, invasive breast tumor phenotype [31, 32]. In this study, we observed that levels of TNF-α were significantly higher in breast cancer patients than controls.

Interleukins are a group of cytokines produced by leukocytes. In comparison to matched normal breast tissue, breast tumors produce significantly increased levels of IL-6, and these levels also increase proportionately with higher tumor grade [33]. Primary human breast tumor cells and human breast cancer cell lines can produce autocrine IL-6, suggesting that carcinoma cells may be the source of the increased levels of IL-6 in serum and tumors [34, 35]. Preliminary studies have indicated that inflammatory cytokine interleukin-8 (IL-8) can be used as a prognostic marker for breast cancer. Serum levels of IL-8 correlate positively with metastatic breast cancer and enhanced expression of IL-8 is associated with poor prognosis of ER negative breast cancer, but not of ER positive breast cancer cases [36]. Several studies have explored the independent negative impact of high levels of IL-6 or IL-8 on prognosis in patients with breast cancer [37]. Our study indicates that higher concentrations of IL-6 and IL-8 were positively associated with increased risk of breast cancer, which is consistent with previous study [38].

Resistin, is also known as adipose tissue-specific secretory factor. It is a 12 kDa cysteine rich polypeptide found in inflammatory zone 3, and belongs to a small family of secreted protein [39]. It is thought that resistin probably upregulates the pro-inflammatory pathways via the NF-κB system [40]. Clinical studies have found that high levels of resistin were associated with the increased risk of breast cancer, and this relationship was independent of age, histological grade, BMI, serum glucose levels, and/or menopause [41].

Visfatin plays a significant role in the nicotinamide adenine dinucelotide (NAD) dependent enzymatic activity which affects various biological responses essential for cell survival [42]. Obese women have lower levels of serum visfatin [43]. Previous studies have reported higher serum visfatin levels in breast cancer patients than controls [44]. *In vitro* studies have shown that visfatin promotes cell proliferation in breast cancer via stimulation of cell cycle progression and increased expression of genes which play important roles in angiogenesis and metastasis [45]. Consistent with previously published studies, our meta-analysis has also confirmed that visfatin levels were positively associated with occurrence of breast cancer.

This study compared for the first time, the concentrations of circulating adipokines between breast cancer patients and controls among different races. As shown in Figure 3, lower adiponectin levels and higher resistin levels were present in Asian cases than controls. However, there was no significant difference in adiponectin and resistin levels between cases and controls among non-Asians. This difference might be explainable by difference in the expression of genes regulating adipogenesis and lipogenesis in adipose tissue [46].

In this study we found that serum levels of all adipokines were significantly associated with incidence breast cancer, whereas no association could be established with plasma levels. However, there is no explanation for this difference at this stage. These results indicate that measurement of serum adipokine levels may be a more reliable predictor of breast cancer. Circulating adipokine levels are most often measured by ELISA, or RIA or Multiplex assay and the first is the most sensitive out of these three. Our results indicate that upon estimation by ELISA, the adipokines show an association with risk of occurrence of breast cancer.

In our meta-analysis, we investigated the correlation between adipokine levels and some traditional prognostic factors of breast cancer such as menopausal status, nodal status, molecular subtypes and TNM stages. Our subgroup analysis suggested that adiponectin, leptin and resistin were associated with incidence of breast cancer in both pre- and postmenopausal women (Supplementary Figure 6). In addition, menopausal status was significantly associated with leptin levels in breast cancer patients, but was not associated with adiponectin and resistin levels (Table 1). Our results imply that leptin is a more effective indicator of breast cancer risk in postmenopausal women than premenopausal women. Our results also show that circulating leptin levels were significantly higher in ER positive breast cancer patients than ER negative patients, This might have been because of estradiol independent activation of leptin among breast cancer tissues [47]. Our results also show that leptin and TNF-α levels are positively correlated with lymph node metastasis status. Moreover, our results also indicate a significantly positive correlation among leptin, IL-6, TNF-α and HGF and TNM stage, and a significantly negative correlation between adiponectin and TNM stage; suggesting that it should be considered as an important factor in tumor growth. Lower adiponectin levels, higher leptin, IL-6, TNF-α and HGF levels appear to be associated with more advanced stages of malignancy.

It is important to mention here that this is the first meta-analysis investigating the association between various adipokines and risk of breast cancer. However, many limitations of our study need to be considered. First, significant heterogeneity existed among studies, thus results should be interpreted cautiously. Subgroup analysis indicated that detection methods and menopausal status were a potential source of heterogeneity. Age, race and the number of cases and controls in the included studies were the other possible sources of heterogeneity. Second, potential confounders might be present in observational studies, and are the intrinsic limitations of observational study design. Although some included studies had used a matching method to select the control group, the actual adipokine levels might have differed from the detection value because of the defects of detection. Third, this meta-analysis includes some studies with small sample sizes i.e*.* those enrolling less than one hundred cases or controls. We used the pooled means and standard deviations of adipokine measures. Statistical tests also suggest publication bias. Fourth, the studies’s methodology used is a limitation, especially the study’s design. Our meta-analysis is mainly based on observational studies, and the inherent limitations of such studies may influence our findings. Case-control studies are mainly subject to selection bias. Besides, the prospective cohorts were over-represented in the Non-Asian group. Lastly, we only included English and Chinese language studies.

## MATERIALS AND METHODS

### Literature search

A comprehensive literature search was carried out using PubMed, EMBASE and Chinese Biomedical Literature (CBM) databases using the following search criteria, “adipokines”, “leptin”, “adiponectin”, “resistin”, “interleukin 6” or “IL-6”, “Tumor Necrosis Factor alpha” or “TNF-α”, “Hepatocyte Growth Factor” or “HGF”, “Plasminogen Activator Inhibitor 1” or “PAI-1”, “visfatin”, “interleukin 8” or “IL-8”, “SFRP-5”, “lipocalin 2”, “apelin” and “breast cancer”, “obesity” or “obese” published before Sept. 15, 2015. In addition, the reference lists of these publications were manually searched for relevant articles.

### Selection criteria

The eligibility of the identified articles was reviewed independently by two authors (Y.G. and L.C.). The criteria for selection of the eligible articles were as follows: (1) cross-sectional study, or case-control study (retrospective or nested case-control), or cohort study (retrospective or prospective cohort study); (2) studies investigating the adipokine levels between obese and nonobese healthy subjects; (3) studies investigating the association between adipokine level, menopausal status, detection method, study sample, or race with risk of breast cancer.

Studies investigating tissue adipokines among male patients were excluded. In the case of multiple publications reporting the same or overlapping data, only the most recent study, or the study using the largest population, as recommended by Wu *et al.*, were included [48]. Studies reporting the number of participants in groups; and mean and SD of adipokine levels were included, or studies in which these values could be calculated from the reported median and range or could be read from figures [48]. The authors of studies with inadequate data were contacted, and in case of no reply from the authors, the studies were excluded. Additionally, studies evaluating AFRP-5, lipocalin-2 and apelin were also excluded as these had been estimated in less than two studies.

### Quality assessment

Two authors (Y.G. and L.C.) independently evaluated the quality of the selected literature using the Newcastle-Ottawa Scale (NOS) criteria [49]. A “score system” was carried out based on the NOS criteria and total scores ranged from 0 (worst) to 9 (best) for case-control or cohort studies, and 0 (worst) to 4 (best) for cross-sectional study (Supplementary Table 3). Disagreement between assessments was resolved by consensus with the third reviewer (X.L.).

### Data extraction

First, we extracted the publication information (name of first author, year of publication and country), characteristics of participants (mean age of participants, sample size and menopausal status of participants), and outcome information (*i.e.* type and detection method of adipokines measurement, and study sample type), and adipokine levels (means and SDs for each group). Next, for the studies providing only the medians and ranges, we calculated the means and SDs [50]. We imputed the range values wherever the interquartile ranges had been mentioned by using the method described by Wu *et al.* [48] We contacted the authors for the quantitative data if the data had been represented by figures only. In case of failure of response, the figures were interpreted by using Engauge Digitizer 4.1 (M. Mitchell, Engauge Digitizer, http://digitizer.sourceforge.net). This can read exact values by digitizing data points from an image file after manually setting the coordinate axis. Disagreements were resolved by discussion with the third reviewer (X.L.).

### Statistical analysis

The summary statistic of this meta-analysis was calculated by use of the standardized mean difference ( SMD ) and the corresponding 95% CIs. Heterogeneity between articles was assessed by chi-square statistics and expressed as an “*I*^2^ ” value. In the event of substantial heterogeneity (*I*^2^ ≥ 50%), study results were obtained using random effects model analysis. Heterogeneity was identified by visual inspection of the forest plots, by using a standard 2-test and a significance level of +0.1, in view of the low power of such tests. Conversely, the pooled SMD were estimated using the fixed effects model analysis. Subgroup analyses were conducted to explore potential sources of heterogeneity including the detection method, sample types, and, menopausal status of patients in the included studies. Publication bias was evaluated using funnel plot analysis. The statistical analysis was performed using Review Manager 5.3 software (RevMan software, Version 5.3, Cochrane Collaboration, United Kingdom) and a *P* value of less than 0.05 was considered to be significant.

## CONCLUSIONS

In conclusion, obese subjects had lower levels of adiponectin and higher levels of leptin and TNF-αthan non-obese subjects. Also, breast cancer patients had lower concentrations of adiponectin and higher concentrations of leptin, IL-6, IL-8, TNF-α, resistin and visfatin than controls. The patterns of altered levels of adipokine among obese subjects may be considered as specific predictors for risk of breast cancer. The results also show that changes in adipokine levels are significantly associated with increased risk of breast cancer, especially among Asians. Our results suggest the existence of an association of obesity, adipokines and risk of breast cancer in Asian women.

## SUPPLEMENTARY MATERIALS FIGURES AND TABLES








