# Supplementary material for: The association between obesity related adipokines and risk of breast cancer: a meta-analysis
Source: Oncotarget. 2017 May 13;8(43):75389–99. doi: 10.18632/oncotarget.17853 (PMC5650429; doi:10.18632/oncotarget.17853)
Supplement: Supplementary file 1 [file oncotarget-08-75389-s001.pdf]

The association between obesity related adipokines and risk of breast cancer: a meta-analysis

Supplementary Materials

SUPPLEMENTARY REFERENCES

1. Jürimäe J, Jürimäe T, Ring-Dimitriou S, LeMura LM, Arciero PJ, von Duvillard SP. Plasma adiponectin and insulin sensitivity in overweight and normalweight middle-aged premenopausal women. Metabolism. 2009; 58:638–643.
2. Ashley DT, O'Sullivan EP, Davenport C, Devlin N, Crowley RK, McCaffrey N, Moyna NM, Smith D, O'Gorman DJ. Similar to adiponectin, serum levels of osteoprotegerin are associated with obesity in healthy subjects. Metabolism. 2011; 60:994–1000.
3. Rigamonti AE, Sartorio A, Bonomo SM, Giunta M, Grassi G, Perotti M, Cella SG, Müller EE, Pincelli AI. Effect of a somatostatin infusion on circulating levels of adipokines in obese women. Metabolism. 2012; 61:1797–1802.
4. Milewicz A, Jędrzejuk D, Dunajska K, Lwow F. Waist circumference and serum adiponectin levels in obese and non-obese postmenopausal women. Maturitas. 2010; 65:272–275.
5. Kristensen K, Pedersen SB, Vestergaard P, Mosekilde L, Richelsen B. Hormone replacement therapy affects body composition and leptin differently in obese and non-obese postmenopausal women. J Endocrinol. 1999; 163:55–62.
6. Silha JV, Krsek M, Skrha JV, Sucharda P, Nyomba BL, Murphy LJ. Plasma resistin, adiponectin and leptin levels in lean and obese subjects: correlations with insulin resistance. Eur J Endocrinol. 2003; 149:331–335.
7. Adamska A, Nikołajuk A, Karczewska-Kupczewska M, Kowalska I, Otziomek E, Górska M, Strączkowski M. Relationships between serum adiponectin and soluble TNF-a receptors and glucose and lipid oxidation in lean and obese subjects. Acta Diabetol. 2012; 49:17–24.
8. Rönnemaa T, Karonen SL, Rissanen A, Koskenvuo M, Koivisto VA. Relation between Plasma Leptin Levels and Measures of Body Fat in Identical Twins Discordant for Obesity. Ann Intern Med. 1997; 126:26–31.
9. Petrášová D, Bertková I, Petrášová M, Hijová E, Mareková M, Babinská I, Jarcuska P, Pella D, Gecková AM; HepaMeta Team. Biomarkers associated with obesity and overweight in the roma population residing in eastern slovakia. Cent Eur J Public Health. 2014; 22:S18–S21.
10. Kazmi A, Sattar A, Hashim R, Khan SP, Younus M, Khan FA. Serum Leptin values in the healthy obese and non-obese subjects of Rawalpindi. JPMA. 2013; 63:245–248.
11. Karhunen LJ, Lappalainen RI, Vanninen EJ, Kuikka JT, Uusitupa MI. Serum Leptin and Regional Cerebral Blood Flow during Exposure to Food in Obese and Normal-Weight Women. Neuroendocrinology. 1999; 69:154–159.
12. Derosa G, Fogari E, D'Angelo A, Bianchi L, Bonaventura A, Romano D, Maffioli P. Adipocytokine Levels in Obese and Non-obese Subjects: an Observational Study. Inflammation. 2013; 36:914–920.
13. El-Haggar SM, Mostafa TM. Adipokines and biochemical changes in Egyptian obese subjects: possible variation with sex and degree of obesity. Endocrine. 2015; 48:878–885.
14. Ahl S, Guenther M, Zhao S, James R, Marks J, Szabo A, Kidambi S. Adiponectin Levels Differentiate Metabolically Healthy vs Unhealthy Among Obese and Nonobese White Individuals. J Clin Endocrinol Metab. 2015; 100:4172–4180.
15. Van-Dielen FMH, Van- Tveer C, Buurman WA, Greve JW. Leptin and Soluble Leptin Receptor Levels in Obese and Weight-Losing Individuals. J Clin Endocrinol Metab. 2002; 87:1708–1716.
16. Couillard C, Mauriège P, Prud'homme D, Nadeau A, Tremblay A, Bouchard C, Després JP. Plasma Leptin Response to an Epinephrine Infusion in Lean and Obese Women. Obes Res. 2002; 10:6–13.
17. Al Maskari MY, Alnaqdy AA. Correlation between Serum Leptin Levels, Body Mass Index and Obesity in Omanis. Sultan Qaboos Univ Med J. 2006; 6:27–31.
18. Olszanecka−Glinianowicz M, Kocełak P, Janowska J, Skorupa A, Nylec M, Zahorska-Markiewicz B. Plasma visfatin and tumor necrosis factor−alpha (TNF−a) levels in metabolic syndrome. Kardiol Pol. 2011; 69, 8:802–807.
19. Phillips CM, Perry IJ. Does Inflammation Determine Metabolic Health Status in Obese and Nonobese Adults? J Clin Endocrinol Metab. 2013; 98:E1610–E1619.
20. Roytblat L, Rachinsky M, Fisher A, Greemberg L, Shapira Y, Douvdevani A, Gelman S. Raised Interleukin-6 Levels in Obese Patients. Obes Res. 2000; 8:673–675.
21. Khaodhiar L, Ling PR, Blackburn GL, Bistrian BR. Serum Levels of Interleukin-6 and C-Reactive Protein Correlate With Body Mass Index Across the Broad Range of Obesity. JPEN J Parenter Enteral Nutr. 2004; 28:410–415.
22. Yesilbursa D, Serdar A, Heper Y, Sarac M, Coskun S, Kazazoglu AR, Cordan J. The effect of orlistat- induced weight loss on interleukin-6 and C-reactive protein levels in obese subjects. Acta Cardiol. 2005; 60:265–269.

23. Koebnick C, Wagner K, Garcia KL, Gruendel S, Lahmann PH, Weickert MO, Möhlig M, Harsch IA, Einig C, Speth M, Katz N, Trippo U, Zunft HJ. Increase in serum resistin during weight loss in overweight subjects is related to lipid metabolism. Int J Obes (Lond). 2006; 30:1097–1103.
24. Anderlová K, Křemen J, Doležalová R, Housová J, Haluzíková D, Kunesová M, Haluzík M. The Influence of Very-Low-Calorie Diet on Serum Leptin, Soluble Leptin Receptor, Adiponectin and Resistin Levels in Obese Women. Physiol. Res. 2006; 55:277–283.
25. Khanna S, Mali AM. Evaluation of tumor necrosis factor-α (TNF-α) levels in plasma and their correlation with periodontal status in obese and non-obese subjects. J Indian Soc Periodontol. 2010; 14:217–221.
26. Rodríguez-Morán M, Guerrero-Romero F. Elevated concentrations of TNF-alpha are related to low serum magnesium levels in obese subjects. Magnesium Research. 2004; 17:189–196.
27. Alokail MS, Al-Daghri N, Abdulkareem A, Draz HM, Yakout SM, Alnaami AM, Sabico S, Alenad AM, Chrousos GP. Metabolic syndrome biomarkers and early breast cancer in Saudi women: evidence for the presence of a systemic stress response and/or a pre-existing metabolic syndrome-related neoplasia risk? BMC Cancer. 2013; 13:54.
28. Al Awadhi SA, Al Khaldi RM, Rammah TA, Kapila K, Mojiminiyi OA. Associations of adipokines & insulin resistance with sex steroids in patients with breast cancer. Indian J Med Res. 2012; 135:500–505.
29. Ahmed SDH, Khanam A, Sultan N, Idrees F, Akhter N. Serum Adiponectin Level Association with Breast Cancer Risk: Evidence from a Case-Control Study. Asian Pacific J Cancer Prev. 2015; 16:4945–8.
30. Assiri AMA, Kamel HFM, Hassanien MFR. Resistin, Visfatin, Adiponectin, and Leptin: Risk of Breast Cancer in Pre- and Postmenopausal Saudi Females and Their Possible Diagnostic and Predictive Implications as Novel Biomarkers. Dis Markers. 2015; 9.
31. Cust AE, Stocks T, Lukanova A, Lundin E, Hallmans G, Kaaks R, Jonsson H, Stattin P. The influence of overweight and insulin resistance on breast cancer risk and tumour stage at diagnosis: a prospective study. Breast Cancer Res Treat. 2009; 113:567–576.
32. Gross AL, Newschaffer CJ, Hoffman-Bolton J, Rifai N, Visvanathan K. Adipocytokines, Inflammation, and Breast Cancer Risk in Postmenopausal Women: A prospective study. Cancer Epidemiol Biomarkers Prev. 2013; 22/7:1319–1324.
33. Gulcelik MA, Colakoglu K, Dincer H, Dogan L, Yenidogan E, Gulcelik NE. Associations between Adiponectin and Two Different Cancers: Breast and Colon. Asian Pacific J Cancer Prev P. 2012; 13:395–8.
34. Gunter MJ, Wang T, Cushman M, Xue X, Wassertheil-Smoller S, Strickler HD, Rohan TE, Manson JE, McTiernan A, Kaplan RC, Scherer PE, Chlebowski RT, Snetselaar L et al. Circulating Adipokines and Inflammatory Markers and Postmenopausal Breast Cancer Risk. J Natl Cancer Inst. 2015; 107/9:djv169.
35. Guo M, Duan X, Cui S, Tian FG, Cao XC, Geng CZ, Fan ZM, Wang X, Wang S, Jiang HC, Zhang JG, Jin F, Tang JH et al. Circulating High-Molecular-Weight (HMW) Adiponectin Level Is Related with Breast Cancer Risk Better than Total Adiponectin: A Case-Control Study. PLOS ONE. 2015;12:0129246.
36. Han Y, Zhang E, Cao W. The clinical significance of determination of adiponectin for the patients of breast cancer. Shaanxi Journal of medicine 2007; 36/10:1381–3.
37. Hancke K, Grubeck D, Hauser N, Kreienberg R, Weiss JM. Adipocyte fatty acid-binding protein as a novel prognostic factor in obese breast cancer patients. Breast Cancer Res Treat. 2010; 119:367–377.
38. Kang JH, Yu BY, Youn DS. Relationship of serum adiponectin and resistin levels with breast cancer risk. J. Korean Med Sci. 2007; 22:117–121.
39. Körner A, Pazaitou-Panayiotou K, Kelesidis T, Kelesidis I, Williams CJ, Kaprara A, Bullen J, Neuwirth A, Tseleni S, Mitsiades N, Kiess W, Mantzoros CS. Total and High-Molecular-Weight Adiponectin in Breast Cancer: In Vitro and in Vivo Studies. J Clin Endocrinol Metab. 2007; 92:1041–1048.
40. Kim BK, Lee JW, Park PJ, Shin YS, Lee WY, Lee KA, Ye S, Hyun H, Kang KN, Yeo D, Kim Y, Ohn SY, Noh DY, et al. The multiplex bead array approach to identifying serum biomarkers associated with breast cancer. Breast Cancer Res. 2009; 11:R22.
41. Miyoshi Y, Funahashi T, Kihara S, Taguchi T, Tamaki Y, Matsuzawa Y, Noguchi S. Association of Serum Adiponectin Levels with Breast Cancer Risk. Clin Cancer Res. 2003; 9:5699–5704.
42. Minatoya M, Kutomi G, Asakura S, Otokozawa S, Sugiyama Y, Ohnishi H, Akasaka H, Miura T, Mori M, Hirata K. Relationship of serum isoflavone, insulin and adiponectin levels with breast cancer risk. Breast Cancer. 2015; 22:452–461.
43. Mantzoros C, Petridou E, Dessypris N, Chavelas C, Dalamaga M, Alexe DM, Papadiamantis Y, Markopoulos C, Spanos E, Chrousos G, Trichopoulos D. Adiponectin and Breast Cancer Risk. J Clin Endocrinol Metab. 2004; 89/3:1102–1107.
44. Ollberding NJ, Kim Y, Shvetsov YB, Wilkens LR, Franke AA, Cooney RV, Maskarinec G, Hernandez BY, Henderson BE, Le Marchand L, Kolonel LN, Goodman MT. Prediagnostic Leptin, Adiponectin, C-reactive Protein and the Risk of Postmenopausal Breast Cancer. Cancer Prev Res (Phila). 2013; 6/3:188–195.
45. Panis C, Herrera ACSA, Aranome AMF, Victorino VJ, Michelleti PL, Morimoto HK, Cecchini AL, Simão AN, Cecchini R. Clinical insights from adiponectin analysis in breast cancer patients reveal its anti-inflammatory

properties in non-obese women. Mol Cell Endocrino. 2014; 382:190–196.

46. Shahar S, Salleh RM, Ghazali AR, Koon PB, Mohamud WN. Roles of Adiposity, Lifetime Physical Activity and Serum Adiponectin in Occurrence of Breast Cancer among Malaysian Women in Klang Valley. Asian Pacific J Cancer Prev. 2010; 11:61–66.
47. Santillán-Benítez JG, Mendieta-Zerón H, Gómez-Oliván LM, Torres-Juárez JJ, González-Bañales JM, Hernández-Peña LV, Ordóñez-Quiroz A. The Tetrad BMI, Leptin, Leptin/Adiponectin (L/A) Ratio and CA 15-3 are Reliable Biomarkers of Breast Cancer. J Clin Lab Anal. 2013; 27:12–20.
48. Tworoger SS, Eliassen AH, Kelesidis T, Colditz GA, Willett WC, Mantzoros CS, Hankinson SE. Plasma Adiponectin Concentrations and Risk of Incident Breast Cancer. J Clin Endocrinol Metab. 2007; 92/4:1510–1516.
49. Zhong XC, Fu ZQ, Song LY, Li YD, Kong XS. Changes and clinical significance of serum adiponectin level in patients with breast cancer. Chin J of Clinical Rational Drug Use. 2013; 6:111–112.
50. Wang Y, Zhang S. Correlation of serum adiponectin, body mass index and lipid metabolic disorder and breast cancer. Chin J Lab Diagn. 2013; 17/5:888–891.
51. Yang L, Hou G. Observation and analysis of Lp(a), adiponectin and NO in patients with mammary adenocarcinoma. Ningxia Med J. 2006; 28/9:676–677.
52. Zhang J, Ma Z, Yu L, Liu LY, Wang F, Guo MM, Wang F, Yu ZG. Associations between breast cancer and serum levels of adiponectin and leptin. J of Shandong University (Health Sciences) 2012; 50/6:110–3.
53. Aliustaoglu M, Bilici A, Gumus M, Colak AT, Baloglu G, Irmak R, Seker M, Ustaalioglu BB, Salman T, Sonmez B, Salepci T, Yaylaci M. Preoperative serum leptin levels in patients with breast cancer. Med Oncol. 2010; 27:388–391.
54. Chen J, Wang Q, Liu Y, Wang W. Clinical value of serum leptin RIA in patients with breast cancer. J of Radioimmunology 2011, 24/1:34.
55. Coskun U, Günel N, Toruner FB, Sancak B, Onuk E, Bayram O, Cengiz O, Yilmaz E, Elbeg S, Ozkan S. Serum leptin, prolactin and vascular endothelial growth factor (VEGF) levels in patients with breast cancer. Neoplasma. 2003; 50/1:41–6.
56. Gao J, Zhang J, Dong Q. Clinical significance of detection of serum leptin, insulin-like growth factor-1 and tumor necrosis factor α in breast cancer patients. Laboratory Medicine. 2005; 20/1:28–29.
57. Geisler J, Haynes B, Ekse D, Dowsett M, Lønning PE. Total body aromatization in postmenopausal breast cancer patients is strongly correlated to plasma leptin levels. J Steroid Biochem 2007 104:27–34.
58. Gu F, Kraft P, Rice M, Michels KB. Leptin and leptin receptor genes in relation to premenopausal breast cancer incidence and grade in Caucasian women. Breast Cancer Res Treat. 2012; 131/1:17–25.
59. Han C, Zhang HT, Du L, Liu X, Jing J, Zhao X, Yang X, Tian B. Serum Levels of Leptin, Insulin, and Lipids in Relation to Breast Cancer in China. Endocrine. 2005; 26/1:19–24.
60. Harris HR, Tworoger SS, Hankinson SE, Rosner BA, Michels KB. Plasma leptin levels and risk of breast cancer in premenopausal women. Cancer Prev Res (Phila) 2011; 4/9:1449–1456.
61. Huang X, Jin W, Pan M. Determination of Serum Leptin and Vascular Endothelial Growth Factor (VEGF) Contents in Patients with Breast Cancer. J of Radioimmunology. 2006; 19/4:267–269.
62. Jiang D, Chen J, Sun J, Xue M, Hu BN, Hu SB. Serum Leptin and Soluble Leptin Receptor (sLR) Levels in Patients with Breast Carcinoma. J of Radioimmunology. 2006; 19/5:382–383.
63. Ji Y, Wang X, Liu Z. Clinical Significance of Determination of Changes of Serum Leptin, TNF-α and STNFR Contents After Chemotherapy in Patients with Breast Cancer. J of Radioimmunology. 2009; 22/4:336–337.
64. Li C, Liu W, Sun H, Zhou JF. Study on the Plasma Leptin Level and Leptin mRNA Expression in Cancerous Breast Tissue in Patients with Breast Carcinoma Complicated with Obesity. J of Radioimmunology. 2006; 19/5:353–355.
65. Liu CL, Chang YC, Cheng SP, Chern SR, Yang TL, Lee JJ, Guo IC, Chen CP. The Roles of Serum Leptin Concentration and Polymorphism in Leptin Receptor Gene at Codon 109 in Breast Cancer. Oncology. 2007; 72:75–81.
66. Liu Y, Xu H, Deng Z, Yan GT. The Clinical Significance of Defermination of serum Leptin, C-reactive protein and Alpha 1-antitrypsin Levels in Patients with Breast Cancer. J of Radioimmunology. 2010; 23/3:243–245.
67. Liu DL, Nan YG, Liu JX, Gu D, Liu W. Clinical Significance of Determination of Changes of Plasma Leptin and Serum CA15-3, IL-8, hs-CRP Levels Both Before and After Operation in Patients with Breast Cancer. J of Radioimmunology. 2012; 25/3:257–258.
68. Lv XJ, Gao XL, Chen X, Wang Y. Research about serum insulin, leptin, C reactive protein in breast cancer combined with diabetes mellitus patients. J of Taishan Medical College. 2014; 35/1:15–16.
69. Mantzoros CS, Bolhke K, Moschos S, Cramer DW. Leptin in relation to carcinoma in situ of the breast: a study of pre-menopausal cases and controls. Int J Cancer. 1999; 80:523–526.
70. Mohammadzadeh G, Ghaffari MA, Bafandeh A, Hosseini SM, Ahmadi B. The Relationship between -2548 G/A Leptin Gene Polymorphism and Risk of Breast Cancer and Serum Leptin Levels in Ahvazian Women. Iran J Cancer Prev. 2015; 2:100–8.
71. Macciò A, Madeddu C, Gramignano G, Mulas C, Floris C, Massa D, Astara G, Chessa P, Mantovani G. Correlation of body mass index and leptin with tumor size and stage of disease in hormone-dependent postmenopausal breast cancer: preliminary results and therapeutic implications. J Mol Med. 2010; 88:677–686.
72. Ozet A, Arpaci F, Yilmaz ML, Ayta H, Ozturk B, Komurcu S, Yavuz AA, Tezcan Y, Acikel C. Effects of Tamoxifen on

the Serum Leptin Level in Patients with Breast Cancer. Jpn J Clin Oncol. 2001; 31/9:424–427.

73. Pazaitou-Panayiotou K, Kelesidis T, Kelesidis I, Kaprara A, Blakeman J, Vainas I, Mpousoulegas A, Williams CJ, Mantzoros C. Growth hormone-binding protein is directly and IGFBP-3 is inversely associated with risk of female breast cancer. Eur J Endocrinol. 2007; 156:187–194.
74. Stattin P, Söderberg S, Biessy C, Lenner P, Hallmans G, Kaaks R, Olsson T. Plasma leptin and breast cancer risk: a prospective study in northern Sweden. Breast Cancer Res Treat. 2004; 86:191–196.
75. Wang X, Hao L, Sun D. Clinical Significance of Detection of Serum Leptin, Insulin-Like Growth Factor-1 and Tumor Necrosis Factor α in Breast Cancer Patients. Acta Acad Med Weifang. 2005; 27/3:188–189.
76. Wang Y, Yao W, Wang BZ, Li TJ, Cao CY, Chen CC, Lu XQ, Huang QX. The expression of leptin and soluble leptin receptor in breast cancer patients serum and the clinical significance. Int J Lab Med. 2015; 36/10:1341–1343.
77. Woo HY, Park H, Ki CS, Park YL, Bae WG. Relationships among serum leptin, leptin receptor gene polymorphisms, and breast cancer in Korea. Cancer Lett. 2006; 237:137–142.
78. Wu MH, Chou YC, Chou WY, Hsu GC, Chu CH, Yu CP, Yu JC, Sun CA. Circulating levels of leptin, adiposity and breast cancer risk. Brit J Cancer. 2009; 100:578 – 582.
79. Yu H, Zhu W, Xu S. Clinical Significance of Determination of Leptin Levels in Patients with Breast Cancer. J of Radioimmunology. 2005; 18/5:327–329.
80. Zhang H, Huang P, Fu M. Clinical Significance of Determination the Changes on Plasma Leptin Serum PRL, E2 and TSGF Levels After Operation in Patients with Breast Cancer. J of Radioimmunology. 2013; 26/6:797–799.
81. Zhu L. The correlation analysis between breast cancer and serum leptin level. China Mdeical Herald. 2011; 36/8:158–159.
82. Ahmed OL, Adel AM, Diab DR, Gobran NS. Prognostic Value of Serum Level of lnterleukin-6 and lnterleukin-8 in Metastatic Breast Cancer Patients. Egypt J Immunol. 2006; 13/2:61–68.
83. Benoy I, Salgado R, Colpaert C, Weytjens R, Vermeulen PB, Dirix LY. Serum Interleukin 6, Plasma VEGF, Serum VEGF, and VEGF Platelet Load in Breast Cancer Patients. Clin Breast Cancer. 2002; 2/4:311–315.
84. Hu J, Zhang Y, Zhu B. Correlation of serum VEGF, TNF-OC, IL–6 of patients with breast cancer and prognosis. Modern Oncology. 2015; 23/05:639–642.
85. Hussein MZ, Fikky AA, Bar LA, Attia O. Serum IL-6 and IL-12 Levels in Breast Cancer Patients. Egypt J Immunol. 2004; 11/2:165–170.
86. Kesler S, Janelsins M, Koovakkattu D, Palesh O, Mustian K, Morrow G, Dhabhar FS. Reduced hippocampal volume and verbal memory performance associated with interleukin-6 and tumor necrosis factor-alpha levels in chemotherapy-treated breast cancer survivors. Brain Behav Immun. 2013; 30/0:S109–S116.
87. Kuang Y, Zhang Z, Zhang X. Interleukin-6 and its soluble receptors in human breast cancer. Chin J Oncol. 1998; 20/4:305–307.
88. Li M, Zhang JY, Lin L, Liu JB. Clinical Value of IL-6 and IL-8 in Serum of Cases with Breast Cancer. J Mod Lab Med. 2011; 26/6:134–137.
89. Li XY, Li J, Fan H, Zhang XD, Liu YH. Changes of the levels of CD69, IL-2 and IL-6 in middle and old aged patients with breast cancer before and after operation. Chinese Journal of Gerontology. 2013; 33/4:1790–1791.
90. Li YM, Liu YK, Liu J, Liu Z, Gao X, Wang H, Li WJ. Expressions of multidrug resistanc-associated protein in peripheral blood mononuclear cells in patients with breast cancer. Hebei Medical Journal. 2015; 37/2:176–179.
91. Ling C, Wu J. Clinical Significance of Serum IL-6, IL-1β and TNF α in Patients with Breast Cancer. Labeled Immunoassays & Clin Med. 2014; 21/5:532–534.
92. Liu F. Changes and clinical significance of serum TGFβ1, IL- 6 and IL- 2 levels in patients with breast cancer. Maternal & Child Health Care of China. 2007; 22:2482–2483.
93. Narita D, Seclaman E, Ursoniu S, Ilina R, Cireap N, Anghel A. Expression of CCL18 and interleukin-6 in the plasma of breast cancer patients as compared with benign tumor patients and healthy controls. Rom J Morphol Embryol. 2011; 52/4:1261–1267.
94. Premkumar VG, Yuvaraj S, Vijayasarathy K, Gangadaran SG, Sachdanandam P. Serum Cytokine Levels of Interleukin-1β, -6, -8, Tumour Necrosis Factor-α and Vascular Endothelial Growth Factor in Breast Cancer Patients Treated with Tamoxifen and Supplemented with Co-Enzyme Q10, Riboflavin and Niacin. Basic Clin Pharmacol. 2007; 100:387–391.
95. Qi QG, Li ZY, Wang YE, Zhang SD, Li CN, Xu YY, Yue L. To explore the relationship between the expression of CCL18, IL-6 and TNF-α in the serum of patients with breast cancer. Chongqing Medicine. 2013; 42/10:1095–1099.
96. Soygur H, Palaoglu O, Akarsu ES, Cankurtaran ES, Ozalp E, Turhan L, Ayhan IH. Interleukin-6 levels and HPA axis activation in breast cancer patients with major depressive disorder. Prog Neuro-Psychoph. 2007; 31:1242–1247.
97. Sun YP, Pan GJ, Wang W, Wang YS. The detection of the levels of serum IL-6 in advanced breast cancer patients. China Journal of Cancer Prevention and Treatment. 2000; 7/2:219.
98. Sun X, Bao Y, Bao J. The significance of detecting serum tumor markers and cytokines for patients with breast cancer. China Medicine and Pharmacy. 2011; 1/13:17–20.
99. Tripsianis G, Papadopoulou E, Anagnostopoulos K, Botaitis S, Katotomichelakis M, Romanidis K, Kontomanolis E, Tentes I, Kortsaris A. Coexpression of IL-6 and TNF-α: prognostic significance on breast cancer outcome. Neoplasma. 2014; 61/2:205–212.

100. Wang Z. Clinical Significance of Determination of Changes of Serum CA153, IL- 6, IL- 8 and TNF-α Levels in Patients with Breast Cancer. J of Radioimmunology. 2006; 19/3:189–191.
101. Wang W, Zhu SB, Cui JY, Wang JP. Changes and clinical significance of serum levels of sFas and -6 IL in patients with ovarian cancer and breast cancer. Shandong Medical Journal. 2007; 47/6:53.
102. Yang ZL, Liu JB, Zhang YJ, Li BW. Changes of serum TNF-α, IL-6 and IL-2 levels perioperatively in patients with breast cancer. Chin J Gen Surg. 2001;16/11:678–679.
103. Yang M, Li D, Yu H. Changes and significance of IL-6, IL-8 and TNF-α in serum of breast cancer patients before and after chemotherapy. Maternal & Child Health Care of China. 2015; 30:136–138.
104. Yu L, Wang B, Liu Y. Clinical significance of combined examination of serum IL 6, FA and TNF in patients with breast cancer. J of Radioimmunology. 2004; 17/1:41–42.
105. Sheen-Chen SM, Chen WJ, Eng HL, Chou FF. Serum concentration of tumor necrosis factor in patients with breast cancer. Breast Cancer Res Treat. 1997; 43:211–215.
106. Krajcik RA, Massardo S, Orentreich N. No Association between Serum Levels of Tumor Necrosis Factor-α(TNF-α) or the Soluble Receptors sTNFR1 and sTNFR2 and Breast Cancer Risk. Cancer Epidem Biomar. 2003; 12:945–946.
107. Papadopoulou e, Tripsianis G, Anagnostopoulos K, Tentes I, Kakolyris S, Galazios G, Sivridis E, Simopoulos K, Kortsaris A. Significance of serum tumor necrosis factor-alpha and its combination with HER-2 codon 655 polymorphism in the diagnosis and prognosis of breast cancer. Int J Biol Markers. 2010; 25/3:126–135.
108. Sheen-Chen SM, Liu YW, Eng HL, Chou FF. Serum Levels of Hepatocyte Growth Factor in Patients with Breast Cancer. Cancer Epidemiol Biomarkers Prev. 2005; 14/3:715–717.
109. Ahmed HH, Metwally FM, Mahdy ESM, Shosha WG, Ramadan SS. Clinical value of serum hepatocyte growth factor, B-cell lymphoma-2 and nitric oxide in primary breast cancer patients. Eur Rev Med Pharmaco. 2012; 16:958–965.
110. Wang Q, Ju F, Wang N, Wang YJ. Relationship between Piasm Urokinase-type Plasminogen Activator(uPA) and Plasminogen Activator Inhibitor(PAl-1)Level and Biological Behaviors in the Breast Cancer Patients. J Med Res. 2010; 39/10:44–49.
111. Dalamaga M, Archondakis S, Sotiropoulos G, Karmaniolas K, Pelekanos N, Papadavid E, Lekka A. Could serum visfatin be a potential biomarker for postmenopausal breast cancer? Maturitas. 2012; 71:301–308.
112. Li XY, Tang SH, Zhou XC, Ye YH, Xu XQ, Li RZ. Preoperative serum visfatin levels and prognosis of breast canceramong Chinese women. Peptides. 2014; 51:86–90.

Supplementary Table 1: Characteristics of the studies reported adipokines level with obese and nonobese healthy subjects included in the meta-analysis. See Supplementary_Table_1

Supplementary Table 2: Characteristics of the studies reported adipokines level with breast cancer included in the meta-analysis. See Supplementary_Table_2

Supplementary Table 3: Quality assessment score scale. See Supplementary_Table_3

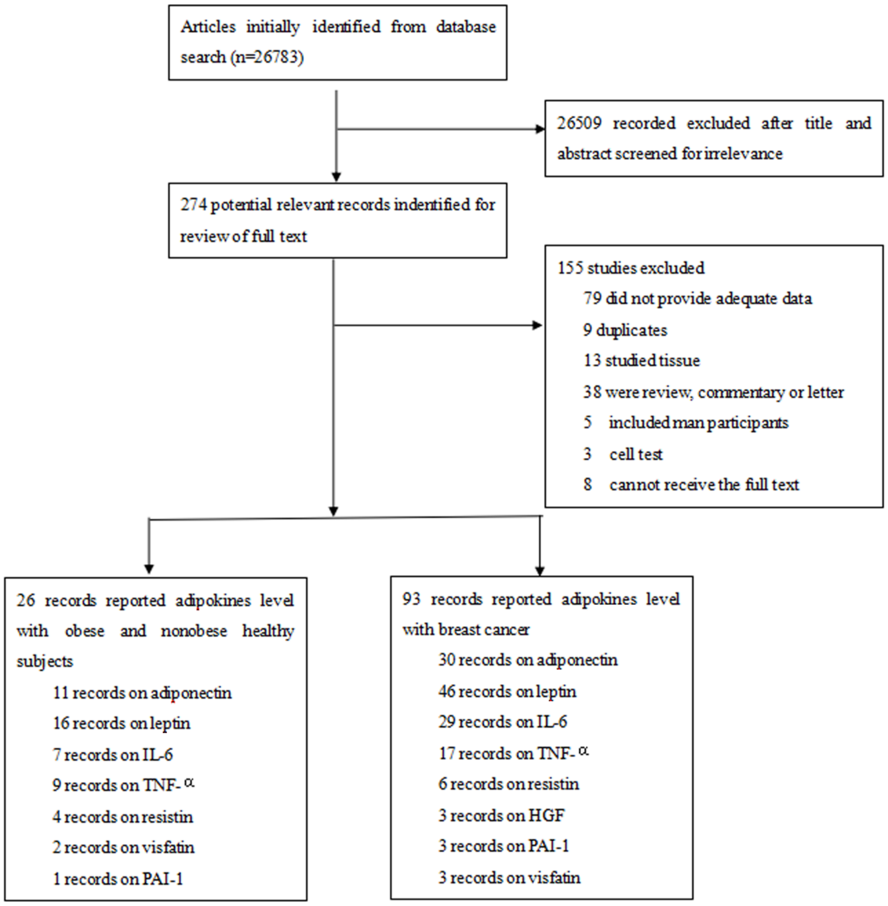

Supplementary Figure 1: Flow diagram depicting the literature search and criteria for selection of the included studies.

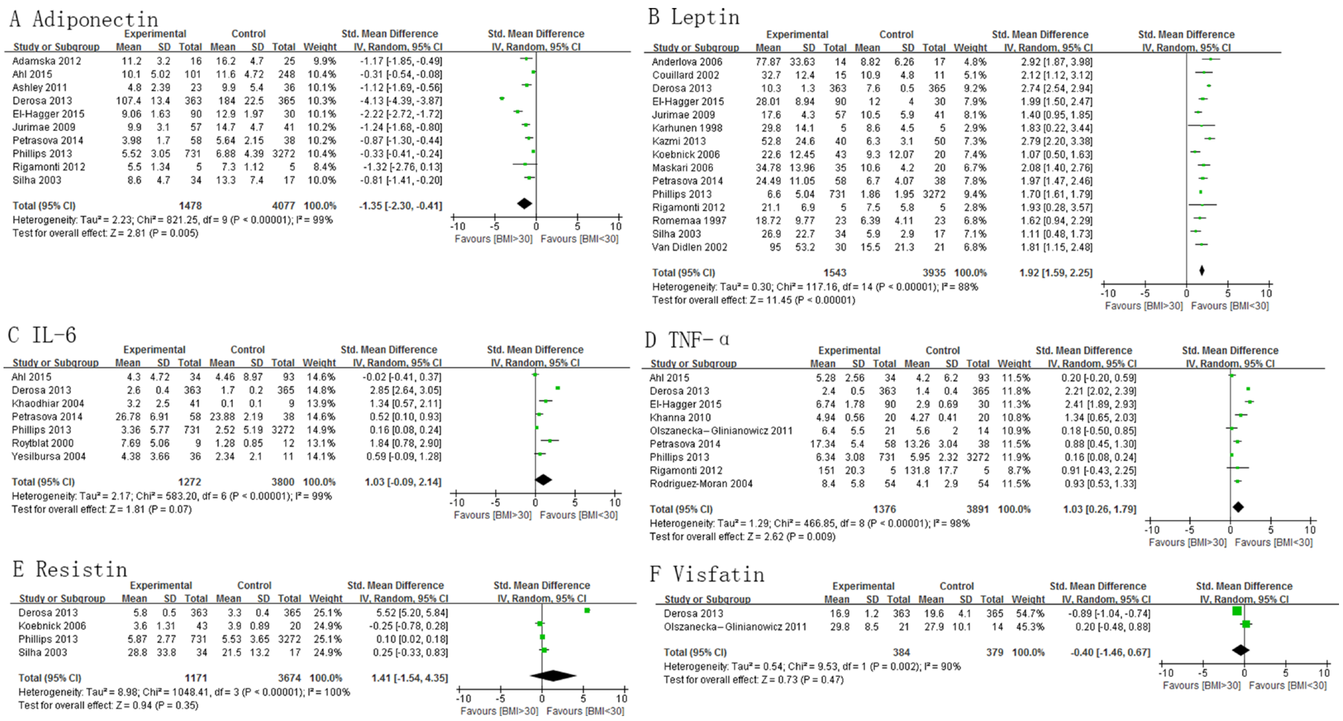

Supplementary Figure 2: Association of adiponectin, leptin, IL-6, TNF-α, resistin and visfatin with BMI > 30 or < 30.

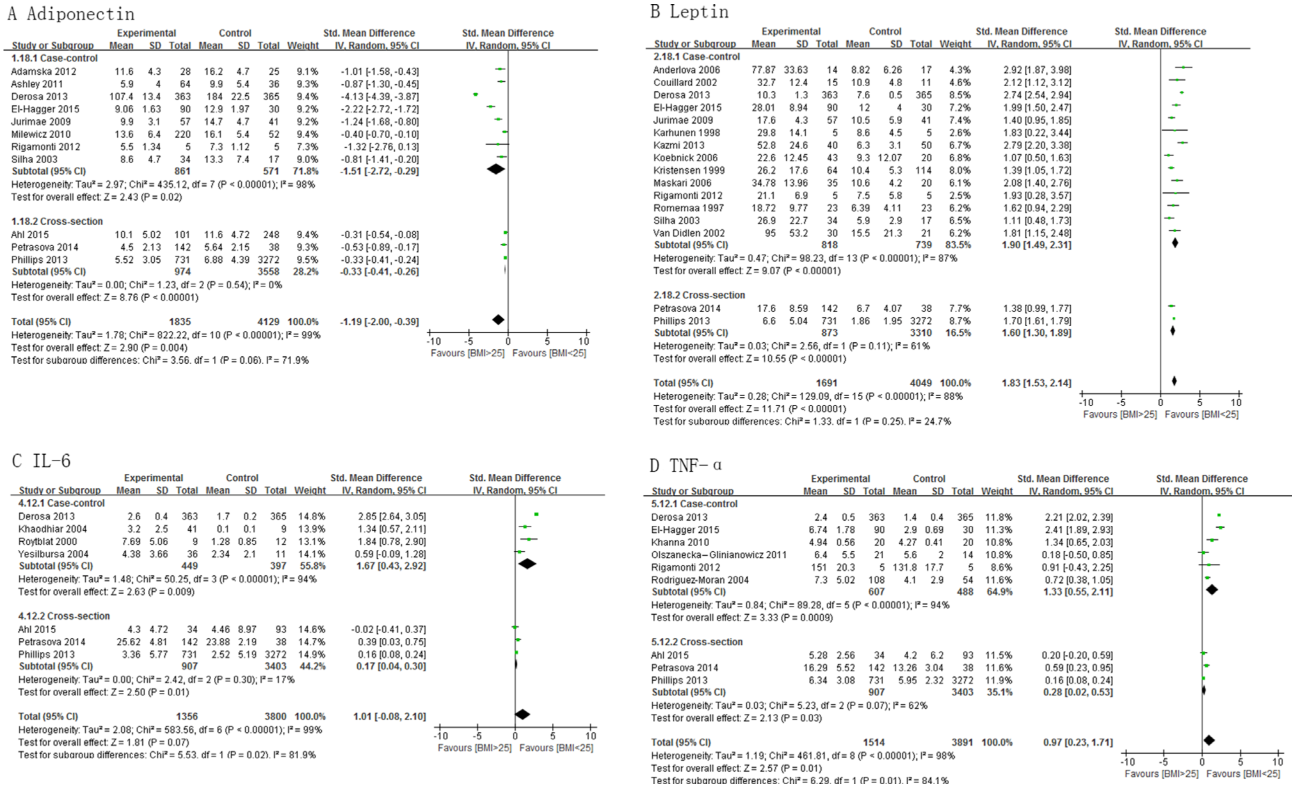

Supplementary Figure 3: Association of adiponectin, leptin, IL-6, and TNF-α with BMI by study method.

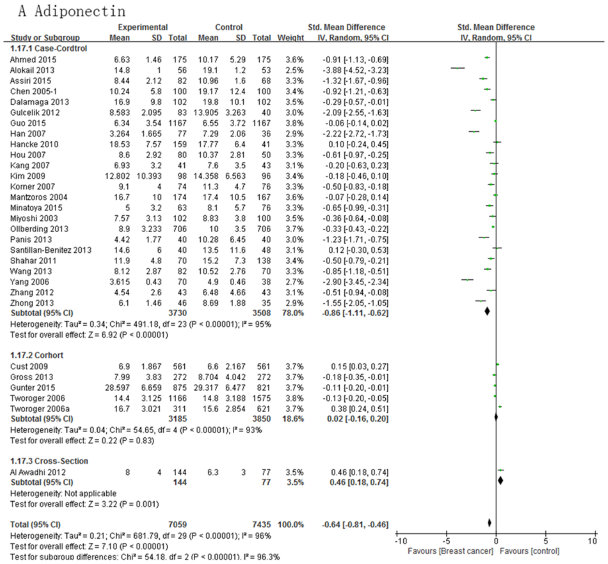

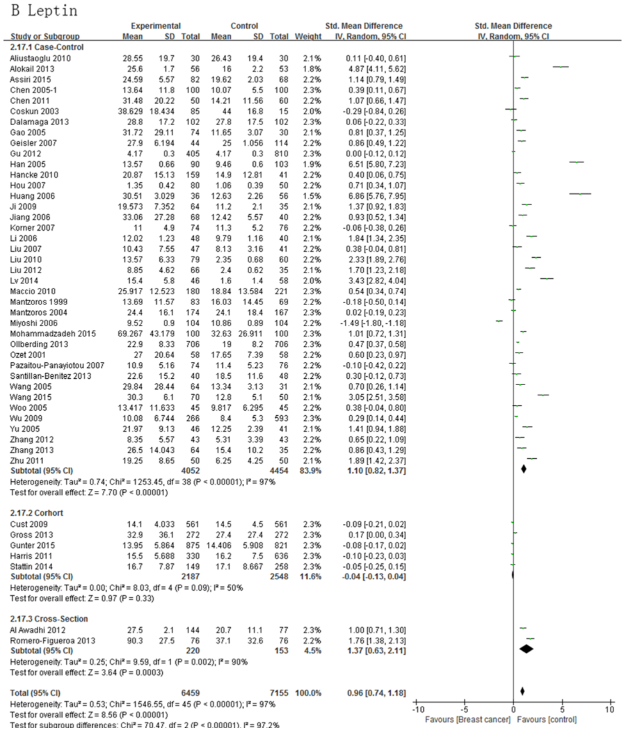

Supplementary Figure 4: Association of adiponectin and leptin with breast cancer risk by study method.

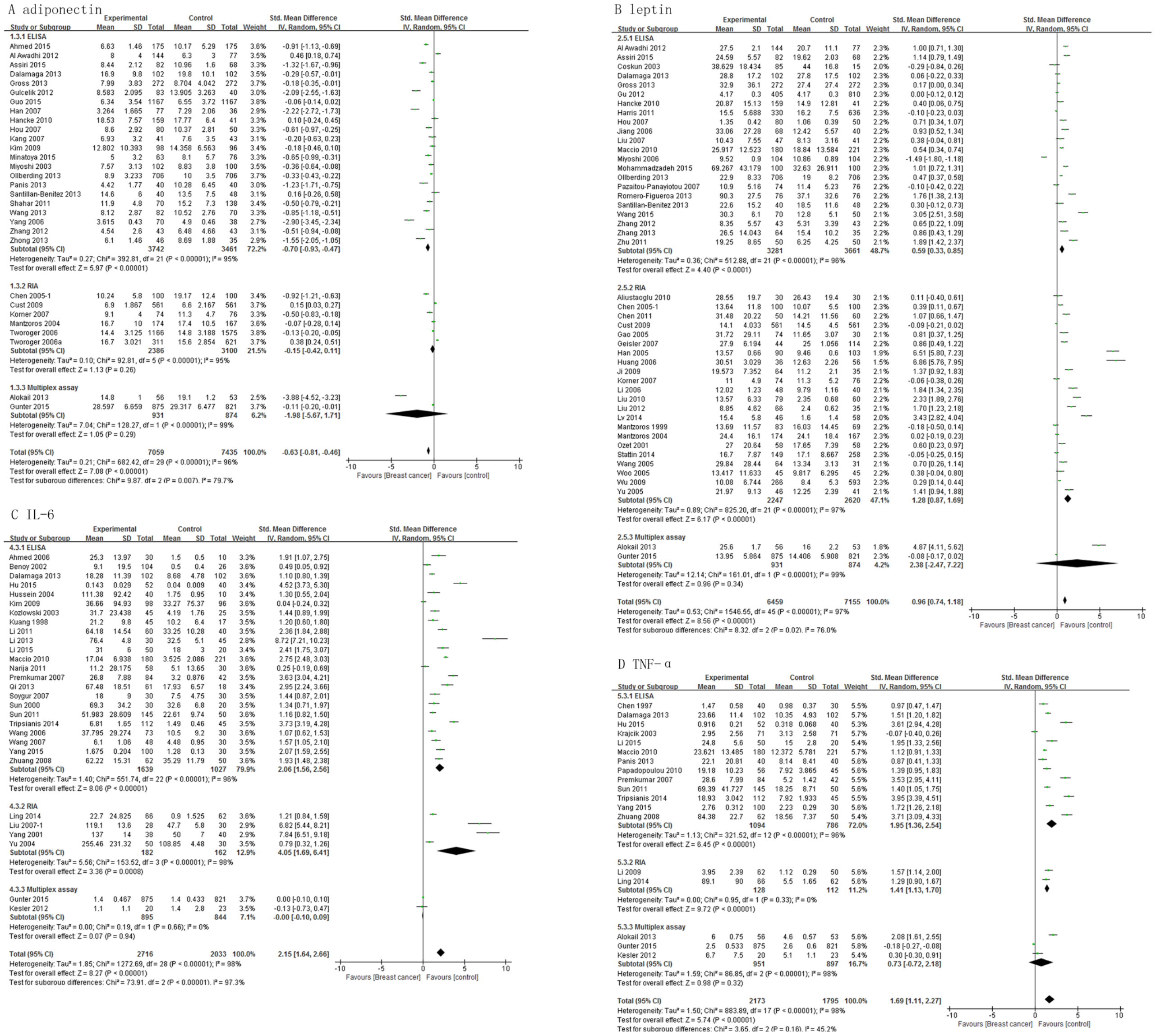

Supplementary Figure 5: Association of adiponectin, leptin, IL-6 and TNF-α with breast cancer risk by assay methods for the analysis of adipokines.

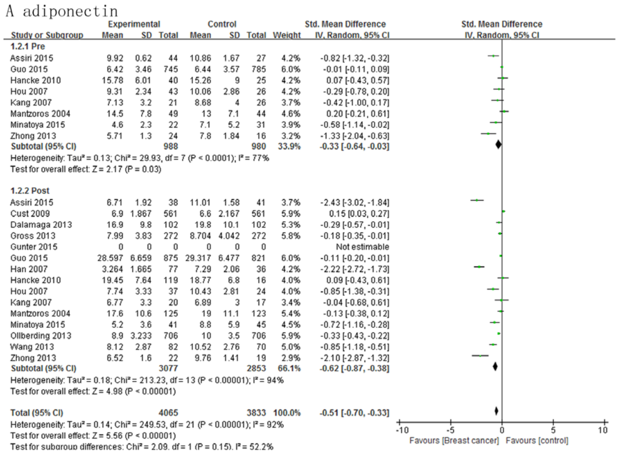

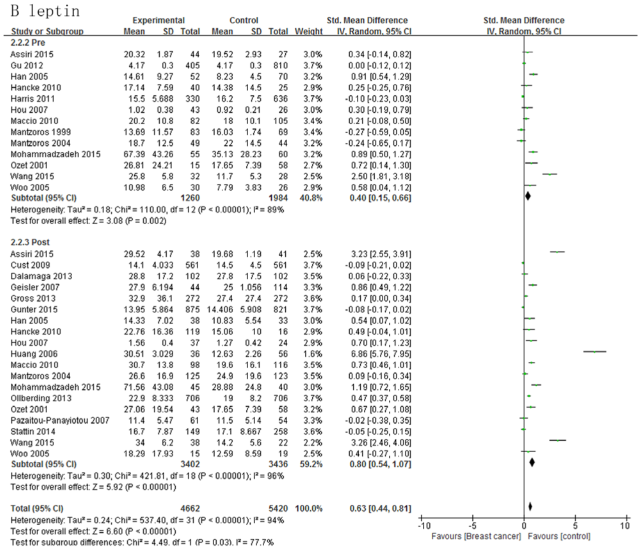

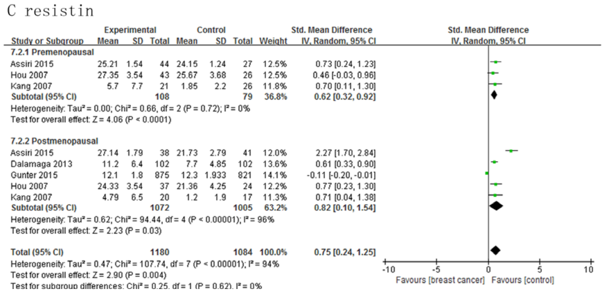

Supplementary Figure 6: Association of adiponectin, leptin and resistin with breast cancer risk by menopausal status subtype.

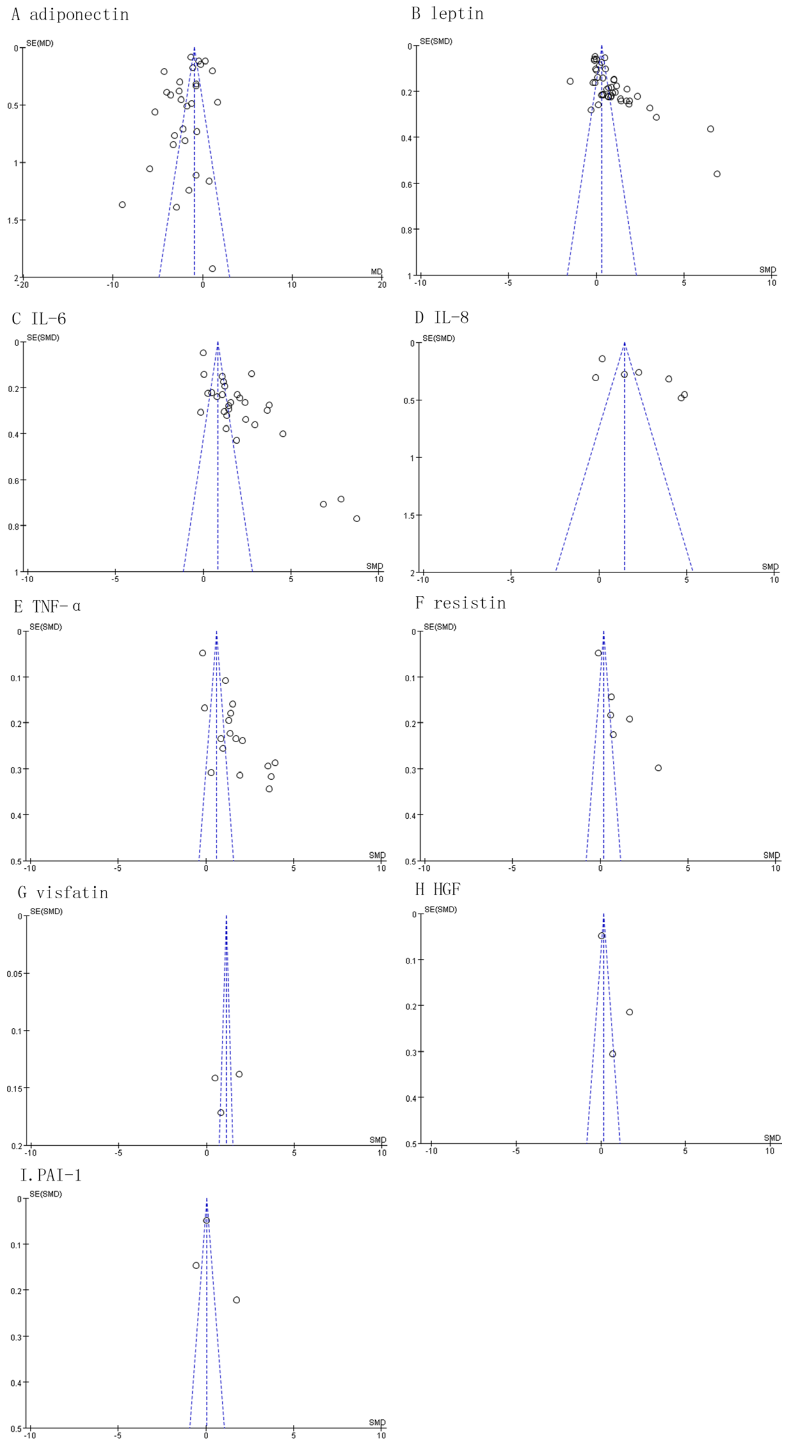

Supplementary Figure 7: Funnel plot analysis to detect publication bias. Each point represents a separate study for the indicated association.
