# Supplementary material for: The association between obesity related adipokines and risk of breast cancer: a meta-analysis
Source: Oncotarget. 2017 May 13;8(43):75389–99. doi: 10.18632/oncotarget.17853 (PMC5650429; doi:10.18632/oncotarget.17853)
Supplement: Supplementary file 2 [file oncotarget-08-75389-s002.doc]

Supplementary Table 1: Characteristics of the studies reported adipokines level with obese and nonobese healthy subjects included in the meta-analysis

| **Ref.** | **First author** | **Year** | **Country/**  **region** | **Study sample** | **Detection method** | **Number of**  **participants** | | **Mean age** | | **NOS** | **Adipocytokines factors** |
| --- | --- | --- | --- | --- | --- | --- | --- | --- | --- | --- | --- |
|  |  |  |  |  |  | **Case** | **Control** | **Case** | **Control** |  |  |
| S1 | Jürimäe | 2009 | Estonia | Plasma | Case-control | 57 | 41 | 44.5 ± 4.9 | 44.5 ± 4.9 | 8 | Adiponectin、Leptin |
| S2 | Ashley | 2011 | Ireland | Serum | Case-control | 41^overweight^  23^obese^ | 36 | 46.7 ± 2.0^overweight^  47.2 ± 2.8^obese^ | 44.4 ± 1.5 | 7 | Adiponectin |
| S3 | Rigamonti | 2012 | Italy | Plasma | Case-control | 5 | 5 | 25.0 ± 4.0 | 20.0 ± 3.0 | 6 | Adiponectin、Leptin、TNF-α |
| S4 | Milewicz | 2010 | Poland | Serum | Case-control | 220 | 52 | NA | NA | 7 | Adiponectin |
| S5 | Kristensen | 1999 | Denmark | Serum | Case-control | 64 | 114 | 50.2 ± 0.3 | 51.5 ± 0.4 | 6 | Leptin |
| S6 | Silha | 2003 | Canada | Plasma | Case-control | 34 | 17 | 45.2±1.0 | 46.6±1.2 | 8 | Adiponectin、Leptin、Resistin |
| S7 | Adamska | 2012 | Poland | Plasma | Case-control | 12^overweight^  16^obese^ | 25 | 25 ± 5.3^overweight^  28.3 ± 7.9^obese^ | 25.1 ± 5.3 | 8 | Adiponectin |
| S8 | Rönnemaa | 1997 | Turku | Plasma | Case-control | 23 | 23 | 45.2±7.6 | 45.2±7.6 | 7 | Leptin |
| S9 | Petrášová | 2014 | Slovakia | Plasma | Cross-section | 84^overweight^  58^obese^ | 38 | 34.85 ± 8.75^overweight^  36.07 ± 8.62^obese^ | 34.85±8.75 | 4 | Adiponectin、Leptin、TNF-α、IL-6 |
| S10 | Kazmi | 2013 | Rawalpindi | Serum | Case-control | 40 | 50 | 34.8±4.6 | 32.7±6.1 | 7 | Leptin |
| S11 | Karhunen | 1998 | Finland | Serum | Case-control | 10 | 12 | 44.8± 9.7 | 40.3 ± 9.7 | 7 | Leptin |
| S12 | Derosa | 2013 | Italy | Plasma | Case-control | 363 | 365 | 35.2±10.4 | 33.6±8.5 | 6 | Adiponectin、Leptin、TNF-α、IL-6、Resistin、Visfatin |
| S13 | El-Haggar | 2015 | Egypt | Serum | Case-control | 30^Class I obese group^  30^ClassII obese group^  30^ClassIII obese group^ | 30 | 32.5±3.08^Class I obese group^  32.4±3.24^ClassII obese group^  32.5±3.10^ClassIII obese group^ | 32±3.02 | 7 | Adiponectin、Leptin、TNF-α |
| S14 | Ahl | 2015 | Wisconsin | Plasma | Cross-section | 1544 | 942 | 48.7±0.4 | 45.5±0.6 | 3 | Adiponectin、TNF-α、IL-6 |
| S15 | Van Dielen | 2002 | Netherlands | Plasma | Case-control | 30 | 21 | 37.2 ± 8.2 | 33.2 ±7.8 | 7 | Leptin |
| S16 | Couillard | 2002 | Canada | Plasma | Case-control | 15 | 11 | 35.6 ± 3.0 | 36.0 ± 3.8 | 7 | Leptin |
| S17 | Maskari | 2006 | Sultanate of Oman | Serum | Case-control | 35 | 20 | 29.4±2.13 | NA | 6 | Leptin |
| S18 | Olszaneck -Glinianowicz | 2011 | Poland | Plasma | Case-control | 21 | 14 | 52.0 ± 9.0 | 38.0 ± 8.0 | 6 | TNF-α、Visfatin |
| S19 | Phillips | 2013 | Ireland | Serum | Cross-section | 731 | 3272 | NA | NA | 4 | Adiponectin、Leptin、TNF-α、IL-6、Resistin、PAI-1 |
| S20 | Roytblat | 2000 | Israel | Serum | Case-control | 9 | 12 | 35.5 ± 9.0 | 32.8 ± 8.2 | 7 | IL-6 |
| S21 | Khanna | 2010 | India | Plasma | Case-control | 20 | 20 | NA | NA | 8 | TNF-α |
| S22 | Khaodhiar | 2004 | Massachusetts | Serum | Case-control | 41 | 9 | 43.1±12.3 | 41.9 ±7.6 | 7 | IL-6 |
| S23 | Yesilbursa | 2004 | Turkey | Serum | Case-control | 36 | 11 | 49.7±8 | 46.2±7 | 7 | IL-6 |
| S24 | Koebnick | 2006 | Germany | Serum | Case-control | 43 | 20 | 53±2 | 39±3 | 7 | Leptin、Resistin |
| S25 | Anderlová | 2006 | Czech Republic | Serum | Case-control | 14 | 17 | NA | NA | 6 | Leptin |
| S26 | Rodriguez-Moran | 2004 | Mexico | Serum | Case-control | 54^overweight^  54^obese^ | 54 | 41.9 ± 14.7^overweight^  43.1 ± 12.4^obese^ | 39.6 ± 14.6 | 7 | TNF-α |
