# Supplementary material for: The association between obesity related adipokines and risk of breast cancer: a meta-analysis
Source: Oncotarget. 2017 May 13;8(43):75389–99. doi: 10.18632/oncotarget.17853 (PMC5650429; doi:10.18632/oncotarget.17853)
Supplement: Supplementary file 3 [file oncotarget-08-75389-s003.doc]

**Supplementary Table 2: Characteristics of the studies reported adipokines level with breast cancer included in the meta-analysis**

| **Ref.** | **First author** | **Year** | **Country/**  **region** | **Study sample** | **Study design** | **Detection method** | **Number of**  **participants** | | **Mean age** | | **Menopausal**  **Status** | | | **NOS** | | **Adipocytokines factors** | | **Adjustment** |
| --- | --- | --- | --- | --- | --- | --- | --- | --- | --- | --- | --- | --- | --- | --- | --- | --- | --- | --- |
|  |  |  |  |  |  |  | **Case** | **Control** | **Case** | **Control** | **Pre.** | **Post** |  | |  | |  | |
| 5 | Dalamaga | 2013 | Greece | Serum | Case-control | ELISA | 102 | 102 | 61.5 (8.2) | 62.8 (8.9) | NA | 204 | 7 | | Adiponectin、Leptin、Resistin、IL-6、TNF-α | | NA | |
| 26 | Chen | 2006 | China | Plasma | Case-control | RIA | 100 | 100 | 49.9±1.0 | 48.9±1.6 | NA | NA | 6 | | Adiponectin、Leptin | | NA | |
| 27 | Romero-Figueroa | 2013 | Mexico | Serum | Cross-section | ELISA | 78 | 78 | 53.2±10.4 | 46.1±10.2 | NA | NA | 4 | | Leptin | | NA | |
| 34 | Zhuang | 2008 | China | Plasma | Case-control | ELISA | 62 | 50 | mean（rang）53（32-93 | 33（20-45） | NA | NA | 7 | | IL-6、TNF-ɑ | | NA | |
| 43 | Hou | 2007 | China | Serum | Case-control | ELISA | 80 | 50 | NA | NA | NA | NA | 7 | | Resistin, Adiponectin and Leptin | | NA | |
| 49 | Miyoshi | 2006 | Japan | Serum | Case-control | ELISA | 104 | 104 | NA | NA | NA | NA | 6 | | Leptin | | NA | |
| S27 | Alokail | 2013 | Kingdom of Saudi Arabia | Serum | Case-control | Multiplex assay | 56 | 53 | 46.4 ± 11.3 | 43.1 ± 7.5 | 78 | 31 | 8 | | Adiponectin、Leptin、TNF-ɑ、Apai-1、Resistin | | NA | |
| S28 | Al Awadhi | 2012 | Kuwait | Plasma | Cross-section | ELISA | 144 | 77 | 50.3 ± 12 | 50.75 ± 12 | 137 | 94 | 3 | | Adiponectin、Leptin | | BMI、Adiponectin、Leptin | |
| S29 | Ahmed | 2015 | Pakistan | Serum | Case-control | ELISA | 175 | 175 | 46.15 ± 0.80 | 44.52 ± 0.80 | 152 | 198 | 7 | | Adiponectin | | NA | |
| S30 | Assiri | 2015 | Saudi Arabia | Plasma | Case-control | ELISA | 82 | 68 | 53.682 ± 13.98 | 52.25 ± 16.62 | 71 | 79 | 8 | | Leptin、Adiponectin、Resistin、Visfatin | | BMI、Adiponectin、Leptin、Resisitin、Visfatin | |
| S31 | Cust | 2009 | Sweden | Plasma | Corhort | RIA | 561 | 561 | 52.5 | NA | NA | NA | 7 | | Adiponectin、Leptin | | NA | |
| S32 | Gross | 2013 | USA | Plasma | Corhort | ELISA | 272 | 272 | 62.6 (9.4) | 62.5 (9.2) | NA | 544 | 8 | | Adiponectin、Leptin | | NA | |
| S33 | Gulcelik | 2012 | Turkey | Serum | Case-control | ELISA | 83 | 40 | 51.4±12.5 | 52.4±10,4 | 41 | 42 | 7 | | Adiponectin | | NA | |
| S34 | Gunter | 2015 | UK | Plasma | Corhort | Multiplex assay | 875 | 839 | 64.0(59.0-69.0) median | 63.0(57.0-69.0) | NA | 1714 | 9 | | Leptin，Adiponectin，Resistin，IL-6，TNF-α，PAI-1，HGF | | NA | |
| S35 | Guo | 2015 | China | Plasma | Case-control | ELISA | 1167 | 1167 | 47.65 (±8.65); | 46.72 (±8.77). | 1530 | 735 | 6 | | Total and HMW adiponectin | | BMI、Adiponectin | |
| S36 | Han | 2007 | China | Serum | Case-control | ELISA | 77 | 36 | 55.6±3.7(non-  metastatic)；  56.2±4.2（metastatic） | 56.3±4.4 | NA | 113 | 7 | | Adiponectin | | NA | |
| S37 | Hancke | 2010 | Germany | Serum | Case-control | ELISA | 159 | 41 | 59.51 (1.0) | 49 (1.7) | 65 | 135 | 7 | | Adiponectin、Leptin | | NA | |
| S38 | Kang | 2007 | Korea | Plasma | Case-control | ELISA | 41 | 43 | 47.4±9.0 | 47.8±6.0 | 47 | 37 | 7 | | Adiponectin、Resistin | | NA | |
| S39 | Körner | 2007 | Greece | Serum | Case-control | RIA | 74 | 76 | 62.5 ± 11.6 | 55.6 ± 11.6 | 33 | 117 | 7 | | Adiponectin、HMW Adiponectin、Leptin | | NA | |
| S40 | Kim | 2009 | Korea | Serum | Case-control | ELISA | 188 | 175 | NA | NA | NA | NA | 6 | | Adiponectin、PAI-1 and IL-6 | | NA | |
| S41 | Miyoshi | 2003 | Japan | Serum | Case-control | ELISA | 102 | 100 | 54.0±1.14 | 52.8 ±1.03 | 97 | 205 | 8 | | Adiponectin | | NA | |
| S42 | Minatoya | 2015 | Japan | Serum | Case-control | ELISA | 63 | 76 | Pre:48.2 ± 9.5;Post:66.1 ± 9.9 | Pre:43.3 ± 12.2;Post:65.2 ± 8.4 | 53 | 86 | 7 | | HMW Adiponectin | | NA | |
| S43 | Mantzoros | 2004 | Greece | Serum | Case-control | RIA | 174 | 167 | NA | NA | 93 | 248 | 6 | | Adiponectin、Leptin | | NA | |
| S44 | Ollberding | 2013 | USA | Serum | Case-control | ELISA | 706 | 706 | 67.8 (7.4) | 67.8 (7.4) | NA | 1412 | 7 | | Adiponectin、Leptin | | BMI、Adiponectin、Leptin | |
| S45 | Panis | 2014 | Brazil | Plasma | Case-control | ELISA | 40 | 40 | median(rang):57.2(32 to 77) | median(rang):57.2(32 to 77) | NA | NA | 8 | | Adiponectin、TNF-α | | NA | |
| S46 | Shahar | 2011 | Malaysia | Serum | Case-control | ELISA | 70 | 138 | 47.3 ± 8.0 | 46.2 ± 6.5 | 242 | 66 | 7 | | Adiponectin | | NA | |
| S47 | Santillan-Benitez | 2013 | Mexico | Serum | Case-control | ELISA | 40 | 40 | 54 ± 10.9 | 41.2 ± 12.9 | NA | NA | 6 | | Adiponectin、Leptin | | NA | |
| S48 | Tworoger | 2006 | US | Plasma | Corhort | RIA | 1477 | 2196 | NHS:57.1 (7.0yr);NHSII:45.4 (4.3) | NHS:58.1 (6.8);NHSII:45.1 (4.3) | 1219 | 2454 | 7 | | Adiponectin | | NA | |
| S49 | Zhong | 2013 | China | Serum | Case-control | ELISA | 46 | 35 | 54.9±6.8 | 55.4±4.8 | 40 | 41 | 8 | | Adiponectin | | NA | |
| S50 | Wang | 2013 | China | Serum | Case-control | SLT-1 | 82 | 70 | mean（SD）55.2（5.1） | 52.9（5.7） | NA | 152 | 7 | | Adiponectin | | NA | |
| S51 | Yang | 2006 | China | Serum | Case-control | ELISA | 35 | 38 | 42-78 | 35-70 | NA | NA | 7 | | Adiponectin、TNF-α | | NA | |
| S52 | Zhang | 2012 | China | Serum | Case-control | ELISA | 43 | 43 | NA | NA | NA | NA | 7 | | Adiponectin、Leptin | | NA | |
| S53 | Aliustaoglu | 2010 | Turkey | Serum | Case-control | RIA | 30 | 30 | median(rang):53(29 to 76) | median(rang):40.4(17 to 69) | 10 | 20 | 8 | | Leptin | | NA | |
| S54 | Chen | 2011 | China | Serum | Case-control | RIA | 50 | 60 | mean（rang）48（26-65） | 37（22-60） | NA | NA | 6 | | Leptin | | NA | |
| S55 | Coskun | 2003 | Turkey | Serum | Case-control | ELISA | 85 | 25 | 51.2± 11.1(non-  metastatic)；  48.5±13.5（metastatic） | 44.5±11.2 | NA | NA | 6 | | Leptin | | NA | |
| S56 | Gao | 2005 | China | Serum | Case-control | RIA | 74 | 30 | rang26-69 | 40-68 | NA | NA | 8 | | Leptin、TNF-ɑ | | NA | |
| S57 | Geisler | 2007 | Norway | Plasma | Case-control | IRMA | 44 | 114 | mean（CI）:69.9(64.2-76.0) | 64.6(63.8-65.5) | NA | 158 | 6 | | Leptin | | NA | |
| S58 | Gu | 2012 | USA | Plasma | Case-control | ELISA | 405 | 810 | 44.8 (4.1) | 44.6 (4.0) | 1215 | NA | 7 | | Leptin | | NA | |
| S59 | Han | 2005 | China | Serum | Case-control | RIA | 90 | 103 | 45.88±9.2 | 46.59 ± 9.60 | 149 | 84 | 8 | | Leptin | | NA | |
| S60 | Harris | 2011 | USA | Plasma | Corhort | ELISA | 330 | 636 | 44.1 (4.0) | 43.6 (3.9) | 966 | NA | 7 | | Leptin | | NA | |
| S61 | Huang | 2006 | China | Serum | Case-control | RIA | 36 | 56 | mean（SD）53.1（3.2） | 58.3（6.4） | NA | 123 | 7 | | Leptin | | NA | |
| S62 | Jiang | 2006 | China | Serum | Case-control | ELISA | 68 | 40 | mean（rang）58.4（49-69） | 56.5（35-67） | NA | NA | 6 | | Leptin | | NA | |
| S63 | Ji | 2009 | China | Serum | Case-control | RIA | 33 | 35 | mean（rang）55.2（32-65） | 58.6（40-70） | NA | NA | 6 | | Leptin、TNF-ɑ | | NA | |
| S64 | Li | 2006 | China | Plasma | Case-control | RIA | 48 | 40 | 53.45（7.69） | 48.23（8.21） | NA | NA | 7 | | Leptin | | NA | |
| S65 | Liu | 2007 | Taiwan | Serum | Case-control | ELISA | 47 | 41 | 50.94±11.24 | 47.5±12.60 | 47 | 40 | 6 | | Leptin | | BMI、Leptin | |
| S66 | Liu | 2010 | China | Serum | Case-control | RIA | 79 | 60 | mean（rang）46.2（24-71） | 45.7（29-76） | NA | NA | 7 | | Leptin | | NA | |
| S67 | Liu | 2012 | China | Plasma | Case-control | RIA | 33 | 35 | NA | NA | NA | NA | 7 | | Leptin | | NA | |
| S68 | Lv | 2014 | China | Serum | Case-control | RIA | 46 | 58 | 50．2±10．1 | 50．4±9．7 | NA | NA | 6 | | Leptin | | NA | |
| S69 | Mantzoros | 1999 | USA | Serum | Case-control | RIA | 83 | 69 | NA | NA | 152 | NA | 8 | | Leptin | | NA | |
| S70 | Mohammadzadeh | 2015 | Iran | Serum | Case-control | ELISA | 100 | 100 | NA | NA | 115 | 85 | 7 | | Leptin | | NA | |
| S71 | Maccio | 2010 | Italy | Serum | Case-control | ELISA | 180 | 221 | Pre:44.1±4.7;  Post:60.5±7.5 | Pre:42.4±8.7;  Post:58.7±8.5 | 187 | 214 | 7 | | Leptin、IL-6、TNF-ɑ | | NA | |
| S72 | Ozet | 2001 | Turkey | Serum | Case-control | RIA | 58 | 58 | 52.03 | 51.83 | NA | NA | 6 | | Leptin | | NA | |
| S73 | Pazaitou-Panayiotou | 2007 | Greece | Serum | Case-control | ELISA | 74 | 76 | 62.5 (11.6) | 55.6 (11.6) | 35 | 115 | 6 | | Leptin | | NA | |
| S74 | Stattin | 2014 | Sweden | Plasma | Corhort | RIA | 149 | 258 | 59.8 (50.1–68.7) | 60.1 (50.1–68.8) | NA | 407 | 7 | | Leptin | | NA | |
| S75 | Wang | 2005 | China | Serum | Case-control | RIA | 64 | 31 | NA | NA | NA | NA | 6 | | Leptin、TNF-ɑ | | NA | |
| S76 | Wang | 2015 | China | Serum | Case-control | ELISA | 70 | 50 | mean（SD）52.0（8.0 | 46.0（7.4） | 60 | 60 | 7 | | Leptin | | NA | |
| S77 | Woo | 2005 | Korea | Serum | Case-control | RIA | 45 | 45 | NA | NA | 56 | 34 | 6 | | Leptin | | NA | |
| S78 | Wu | 2009 | Taiwan | Plasma | Case-control | RIA | 297 | 593 | 49.66±8.72 | 48.71±8.51 | 504 | 352 | 7 | | Leptin | | NA | |
| S79 | Yu | 2005 | China | Serum | Case-control | RIA | 46 | 41 | mean（SD）59.38（6.74） | 61.3（5.59） | NA | NA | 8 | | Leptin | | NA | |
| S80 | Zhang | 2013 | China | Plasma | Case-control | ELISA | 33 | 35 | mean（rang）42.5（32-50） | 43.6（32-52） | NA | NA | 7 | | Leptin | | NA | |
| S81 | Zhu | 2011 | China | Serum | Case-control | ELISA | 50 | 50 | mean（rang）46.5（23-81） | 48.2（22-80） | NA | NA | 6 | | Leptin | | NA | |
| S82 | Ahmed | 2006 | Egypt | Serum | Case-control | ELISA | 30 | 10 | mean（rang）47.1（24-73 | 45.5（40-53 | NA | NA | 7 | | IL-6 | | NA | |
| S83 | Benoy | 2002 | Belgium | Serum | Case-control | ELISA | 104 | 26 | NA | NA | NA | NA | 6 | | IL-6 | | NA | |
| S84 | Hu | 2015 | China | Serum | Case-control | ELISA | 52 | 40 | mean（SD）47.6（10.3） | 45.3±8.1 | NA | NA | 6 | | IL-6、TNF-ɑ | | NA | |
| S85 | Hussein | 2004 | Egypt | Serum | Case-control | ELISA | 40 | 10 | NA | NA | NA | NA | 6 | | IL-6 | | NA | |
| S86 | Kesler | 2012 | USA | Serum | Case-control | Multiplex assay | 42 | 35 | 54.6 (6.5) | 55.5 (9.3) | 26 | 51 | 7 | | IL-6、TNF-ɑ | | NA | |
| S87 | Kozlowski | 2003 | Poland | Serum | Case-control | ELISA | 45 | 25 | 25-79 | NA | NA | NA | 6 | | IL-6 | | NA | |
| S88 | Kuang | 1998 | China | Serum | Case-control | ELISA | 45 | 17 | mean（SD）52.8（12.7） | 30.7（9.0） | NA | NA | 6 | | IL-6 | | NA | |
| S89 | Li | 2011 | China | Serum | Case-control | ELISA | 60 | 40 | 57（27-78） | 33（19-47） | NA | NA | 6 | | IL-6 | | NA | |
| S90 | Li | 2013 | China | Plasma | Case-control | ELISA | 45 | 30 | 61.5±5.4 | NA | NA | NA | 6 | | IL-6 | | NA | |
| S91 | Li | 2015 | China | Serum | Case-control | ELISA | 50 | 20 | 68±8 | 67±7 | NA | NA | 7 | | IL-6、 TNF-α | | NA | |
| S92 | Ling | 2014 | China | Serum | Case-control | RIA | 66 | 62 | mean（SD）47.5（10.1） | 48.1±6.6 | NA | NA | 7 | | IL-6、 TNF-α | | NA | |
| S93 | Liu | 2007-1 | China | Serum | Case-control | RIA | 28 | 30 |  |  |  |  | 6 | | IL-6 | | NA | |
| S94 | Narita | 2011 | Italy | Plasma | Case-control | ELISA | 58 | 30 | median(rang):60(35 to 90) | NA | NA | NA | 7 | | IL-6 | | NA | |
| S95 | Premkumar | 2007 | India | Serum | Case-control | ELISA | 84 | 42 | NA | NA | NA | NA | 6 | | IL-6、 TNF-α | | NA | |
| S96 | Qi | 2013 | China | Serum | Case-control | ELISA | 61 | 18 | median46.3 | NA | NA | NA | 6 | | IL-6 | | NA | |
| S97 | Soygur | 2007 | Turkey | Plasma | Case-control | ELISA | 30 | 30 | 43.0±5.8 | 37.9±9.6 | NA | NA | 7 | | IL-6 | | NA | |
| S98 | Sun | 2000 | China | Serum | Case-control | ELISA | 30 | 20 | 48（35-60） | 20-60 | NA | NA | 6 | | IL-6 | | NA | |
| S99 | Sun | 2011 | China | Serum | Case-control | ELISA | 145 | 50 | mean（rang）47.52（26-70） | 42.17（20-70） | NA | NA | 6 | | IL-6、TNF-ɑ | | NA | |
| S100 | Tripsianis | 2014 | Greece | Serum | Case-control | ELISA | 112 | 45 | 63.41±12.00 | NA | NA | NA | 7 | | IL-6、TNF-ɑ | | NA | |
| S101 | Wang | 2006 | China | Serum | Case-control | ELISA、RIA | 38 | 30 | mean（rang）47.2（32-56） | 46.8(30-57) | NA | NA | 7 | | IL-6、TNF-ɑ | | NA | |
| S102 | Wang | 2007 | China | Serum | Case-control | ELISA | 48 | 30 | median（rang）（33-74） | 18-58 | NA | NA | 6 | | IL-6 | | NA | |
| S103 | Yang | 2001 | China | Serum | Case-control | RIA | 38 | 40 | NA | NA | NA | NA | 6 | | IL-6、TNF-ɑ | | NA | |
| S104 | Yang | 2015 | China | Serum | Case-control | ELISA | 50 | 30 | 49.1±4.7 | 46.3±3.4 | NA | NA | 6 | | IL-6、TNF-ɑ | | NA | |
| S105 | Yu | 2004 | China | Serum | Case-control | RIA | 50 | 30 | mean（rang）54（37-65 | 45-70 | NA | NA | 7 | | IL-6 | | NA | |
| S106 | Sheen-Chen | 1997 | Taiwan | Serum | Case-control | ELISA | 40 | 30 | mean（rang）50（28-68 | NA | NA | NA | 6 | | TNF-α | | NA | |
| S107 | Krajcik | 2003 | USA | Serum | Case-control | ELISA | 142 | 142 | 41.5 (19 –73) | 55.1 (31– 83) | NA | NA | 7 | | TNF-α | | NA | |
| S108 | Papadopoulou | 2010 | Greece | Serum | Case-control | ELISA | 56 | 45 | 61.34±11.64 | 57.20±12.04 | 32 | 69 | 7 | | TNF-α | | NA | |
| S109 | Sheen-Chen | 2005 | Taiwan | Serum | Case-control | ELISA | 124 | 35 | 50.5（31-84） | 50.5（31-84） | NA | NA | 6 | | HGF | | NA | |
| S110 | Ahmed | 2012 | Egypt | Serum | Case-control | ELISA | 44 | 15 | median(rang):36(23-56) | NA | NA | NA | 7 | | HGF | | NA | |
| S111 | Wang | 2010 | China | Plasma | Case-control | ELISA | 88 | 39 | median(rang):52(39-72) | 48（22-78） | NA | NA | 6 | | PAI-1 | | NA | |
| S112 | Dalamaga | 2012 | Greece | Serum | Case-control | ELISA | 103 | 103 | mean（SD）61.5 (8.2) | 62.3 (8.8) | NA | 206 | 7 | | Visfatin | | NA | |
| S113 | Li | 2014 | China | Serum | Case-control | ELISA | 248 | 100 | NA | NA | NA | NA | 8 | | Visfatin | | NA | |
