# Supplementary material for: The association between obesity related adipokines and risk of breast cancer: a meta-analysis
Source: Oncotarget. 2017 May 13;8(43):75389–99. doi: 10.18632/oncotarget.17853 (PMC5650429; doi:10.18632/oncotarget.17853)
Supplement: Supplementary file 4 [file oncotarget-08-75389-s004.doc]

**Supplementary Table 3. Quality assessment score scale**

| **Item** | **Score** |
| --- | --- |
| **1.NOS score scale for case-control studies*** |  |
| **Selection** |  |
| (1) Is the case definition adequate? |  |
| Yes, with independent validation | 1 |
| Yes, e.g. record linkage or based on self reports | 0 |
| No description | 0 |
| (2) Representativeness of the cases |  |
| Consecutive or obviously representative series of cases | 1 |
| Potential for selection biases or not stated | 0 |
| (3) Selection of Controls |  |
| Community controls | 1 |
| Hospital controls | 0 |
| No description | 0 |
| (4) Definition of Controls |  |
| No history of disease (endpoint) | 1 |
| No description of source | 0 |
| **Comarability** |  |
| (1) Comparability of cases and controls on the basis of the design or analysis |  |
| Study controls for the most important factor | 1 |
| Study controls for any additional factor | 1 |
| **Exposure** |  |
| (1) Ascertainment of exposure |  |
| Secure record (e.g. surgical records) | 1 |
| Structured interview where blind to case/control status | 1 |
| Interview not blinded to case/control status | 0 |
| Written self report or medical record only | 0 |
| No description | 0 |
| (2) Same method of ascertainment for cases and controls |  |
| Yes | 1 |
| No | 0 |
| (3) Non-Response rate |  |
| Same rate for both groups | 1 |
| Non respondents described | 0 |
| Rate different and no designation | 0 |
| **2.NOS score scale for cohort studies^†^** |  |
| **Selection** |  |
| (1) Representativeness of the exposed cohort |  |
| Truly representative of the average status in the community | 1 |
| Somewhat representative of the average status in the community | 1 |
| Selected group of users (e.g. nurses, volunteers) | 0 |
| No description of the derivation of the cohort | 0 |
| (2) Selection of the non exposed cohort |  |
| Drawn from the same community as the exposed cohort | 1 |
| Drawn from a different source | 0 |
| No description of the derivation of the non exposed cohort | 0 |
| (3) Ascertainment of exposure |  |
| Secure record (e.g. surgical records) | 1 |
| Structured interview | 1 |
| Written self report | 0 |
| No description | 0 |
| (4) Demonstration that outcome of interest was not present at start of study |  |
| Yes | 1 |
| No | 0 |
| **Comparability** |  |
| (1) Comparability of cohorts on the basis of the design or analysis |  |
| Study controls for the most important factor | 1 |
| Study controls for any additional factor | 1 |
| **Outcome** |  |
| (1) Assessment of outcome |  |
| Independent blind assessment | 1 |
| Record linkage | 1 |
| Self report | 0 |
| No description | 0 |
| (2) Was follow-up long enough for outcomes to occur? |  |
| Yes | 1 |
| No | 0 |
| (3) Adequacy of follow up of cohorts |  |
| Complete follow up - all subjects accounted for | 1 |
| Subjects lost to follow up unlikely to introduce bias | 1 |
| Follow up rate is low and no description of those lost | 0 |
| No statement | 0 |
| **3.NOS score scale for cross-sectional studies^‡^** |  |
| **Selection** |  |
| Representativeness of the participants |  |
| Random sampling | 1 |
| Not random sampling or no description | 0 |
| **Factors** |  |
| Secure record (e.g. surgical records) | 1 |
| Structured interview with blind | 1 |
| Written self report or medical record only | 0 |
| No description | 0 |
| **Comparability** |  |
| Test for comparability or adjust confounding factors | 1 |
| No adjustment | 0 |
| **Non-Response rate** |  |
| Response rate is high enough | 1 |
| Response rate is low or no description | 0 |

*A study can be awarded a maximum of one score for each numbered item within the Selection and Exposure categories. A maximum of two scores can be given for Comparability.

**^†^**A study can be awarded a maximum of one score for each numbered item within the Selection and Outcome categories. A maximum of two scores can be given for Comparability

**^‡^**A study can be awarded a maximum of one score for each item with Selection, Factors, Comparability and Non-response rate.
